# Genomic characterization and insights into the belted coat pattern of a local, reconstituted pig population

**DOI:** 10.1080/10495398.2025.2515462

**Published:** 2025-06-10

**Authors:** Samira Giovannini, Maria Giuseppina Strillacci, Raffaella Milanesi, Caterina Altissimi, Massimo Biagetti, Francesca Maria Sarti

**Affiliations:** a Department of Agricultural, Food and Environmental Sciences, Università degli Studi di Perugia, Perugia, Italy; b Department of Veterinary Medicine and Animal Science, Università degli Studi di Milano, Lodi, Italy; c Department of Veterinary Medicine, Università degli Studi di Perugia, Perugia, Italy; d Istituto Zooprofilattico Sperimentale dell’Umbria e delle Marche-Togo Rosati (IZSUM), Perugia, Italy

**Keywords:** Pig, belted, genomics, local breed, Cinghiato population

## Abstract

The Cinghiato pig population originates from a breeding project aimed at reconstituting an extinct local swine breed, historically depicted in frescoes in Umbria, Central Italy. The selection strategy employed a reconstruction breeding program, choosing mating pairs based on the unique coat phenotype represented in these artworks. Unlike the traditional Cinta Senese breed, Cinghiato pigs exhibit a white belt encircling the trunk, while their forelimbs remain black. This study explores the genetic background of the belted coat pattern observed in the heterogeneous reconstituted population. Twenty-two pigs were genotyped using the Porcine GGP 80K SNP BeadChip. Genetic analyses were conducted to assess population structure and diversity, with comparisons made to other Italian pig breeds and wild boars. Findings reveal moderate genetic diversity within the Cinghiato population. Runs of Homozygosity patterns suggest historical inbreeding events. Moreover, several genomic regions were associated with traits relevant to niche pork production, including feed intake, leg conformation, and fat deposition. Polymorphisms were detected in 10 coat color-related genes (*KIT, MC1R, ASIP, EDNRB, KITLG, MITF, OCA2, PAX3, SOX10*, and *TYRP1*). Although some candidate variants were identified, this preliminary study highlights the need for further research to clarify the genetic mechanisms underlying the phenotypic variability of belted coat patterns.

## Introduction

Livestock breeds of domesticated animals serve as vital connectors between natural ecosystems and human-influenced environments, significantly contributing to global biodiversity and ecosystem services.^1^ However, this essential role is under threat, with 27.22% of the 7,039 existing local livestock breeds facing risk of extinction, according to the Domestic Animal Diversity Information System DAD-IS (https://www.fao.org/dad-is/, accessed 20/04/2024).

This concerning trend has led to the exploration of alternative strategies to ensure the resilience of ecosystems and human activity, including approaches aimed at the reconstruction of phenotypic traits belonging to historically documented but no longer existing populations.^2^

In this context, practices like the *‘reconstruction’*, in line with the *European Regulation 2016/1012,* have been employed to recover ancestral traits.^2^^,^^3^

Such methods, while aligned with traditional selective breeding practices, involve pairing individuals based on specific morphological characteristics in order to recreate phenotypes observed in historical records or iconography. Notable examples include domestic cattle bred to resemble the extinct Auroch (*Bos primigenius*),^4^ or Plains zebras selected to replicate the appearance of the now-extinct quagga (*E. quagga quagga*) (https://www.quaggaproject.org/).

**Figure 1.**
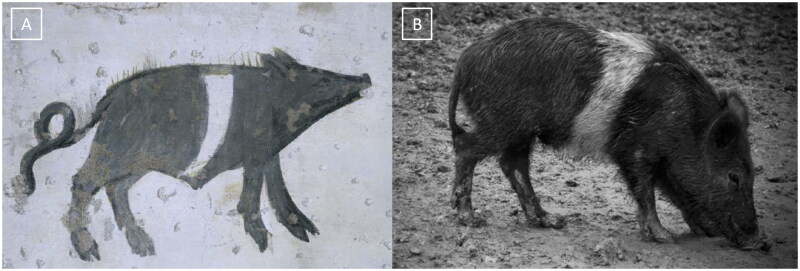
(A) Fresco of a Cinghiato pig from Chiesa Francescana di Santa Maria Assunta a Vallo di Nera, Umbria, Italy, depicting a pig with a belted coat pattern characterized by a white belt across the middle of the trunk, excluding the forelimbs; (B) a Cinghiato gilt from the selection project, exhibiting the same belted coat pattern with a centrally positioned white belt on the trunk that does not encircle the forelimbs (photo of Marco Caffarelli, source https://biodiversita.umbria.parco3a.org/wp-content/uploads/2020/05/Quaderno-Biodiversità_4.pdf).

The subject of this study, the Cinghiato pig population, emerged from such a reconstruction effort, motivated by the discovery of numerous frescoes in central Italy depicting pigs with a distinctive coat pattern. This area has a deep-rooted tradition in pig meat production and processing, and the frescoes—often linked to representations of Sant’Antonio Abate—highlight pigs with a unique belted phenotype (Fig. 1A).^5^

The reconstruction of the Cinghiato population involved crossing Cinta Senese pigs—most resembling the desired phenotype—with hybrids of Cinta Senese and Italian wild boars. Unlike the typical Cinta Senese pattern, frescoes portray pigs with a white belt that excludes the forelimbs, which remain black (Fig. 1B).^5^ Interestingly, as reported by farmers, this trait occasionally appeared in litters resulting from natural matings between domestic sows and wild boars, which may occur in outdoor farming systems. Farmers then selected and bred these individuals to preserve the characteristic appearance.

Today, the Cinghiato remains an unofficial population, maintained by a small number of farms for traditional purposes and niche markets. The unique coat pattern of the Cinghiato pig, beyond its esthetic value represents an important cultural and historical heritage for the region and a peculiarity that deserves investigation of its genetic control.

This study then aims to genomically characterize the Cinghiato pig and to explore the genetic basis of the belted coat pattern. For this last purpose, polymorphisms of ten genes known to be involved in coat color expression (*KIT, MC1R, ASIP, EDNRB, KITLG, MITF, OCA2, PAX3, SOX10*, and *TYRP1*) were considered.

## Materials and methods

### Sampling and genotyping

A total of 22 Cinghiato blood samples were obtained from two selected farms that were part of a PSR project 16.1 (Piani di Sviluppo Rurale, Regione Umbria, 2019–2021). The project aimed to resume a previous attempt made in 2009,^5^ with the goal of developing a conservation plan for a pig population displaying morphological traits closely resembling those depicted in frescoes found in numerous churches across central Italy. These farms were chosen because they historically preserved pigs with wild boar-like morphology for esthetic and meat quality reasons. The animals analyzed in this study matched the characteristics of the ‘*suino cinghiato*’, displaying variations in belt patterns, either encircling the forelimbs and shoulders or positioned around the mid-trunk.^5^ The project’s activities can be seen as a form of reconstruction effort, seeking to restore ancestral traits through controlled breeding. Farms were strategically selected for their similar environments and outdoor breeding systems, and in some cases, genealogical records provided valuable insights into the ancestry and breeding history of the sampled animals.

The DNA extraction from blood was performed using the Quick-DNA™ Miniprep Kit of Zymo Research (Zymo Research Corporation), according to the standard protocol. After a quality check on DNA, the genotyping was done utilizing the BeadChip Porcine-GGP 80K by NEOGEN lab. Unmapped and duplicated SNPs and those on chromosomes different from autosomes were removed from the dataset that resulted then including 63,015 SNP markers. SNPs coordinates were in concordance with Sscrofa 11.1 genome assembly.

### Genetic characterization of Cinghiato population

SNP & Variation Suite (SVS) v8.9 (Golden Helix Inc., Bozeman, MT, USA) software was used to quality check the genotyping data and to filter out SNPs with no assigned position, a minor allele frequency ≥ 0.05. All samples had a call rate greater than 90%. The final SNPs dataset resulted in 40,666 markers (63,015 SNPs before filtering).

SVS software was also employed to:

- estimate the average minor allele frequency (MAF);
- perform a principal component analysis (PCA), the results of which were graphically visualized with the R package ‘ggplot2’;^7^
- calculate the inbreeding coefficient (F_HOM_) for each sample;
- estimate the observed (H_O_) and expected (H_E_) heterozygosity;
- identify runs of homozygosity (ROHs) (63,015 SNPs).

ROHs were identified considering a minimum of 20 SNPs, no heterozygous genotypes neither opposite (i.e., heterozygous) and missing genotypes were allowed, a maximum allowed gap between consecutive SNPs within a ROH set at 1 Mbp and a minimum length of an ROH of 1Mbp, to avoid the detection of common and short ROH across the genome due to linkage disequilibrium (LD). These parameters were chosen in accordance with those used in other studies detecting ROH with similar SNP chip density,^9^ but were more stringent (in terms of missing and heterozygous markers) to reduce false positive detections. ROH sections were categorized into five length classes following the nomenclature in literature:^8^ 1–2, 2–4, 4–8, 8–16, and >16 Mbp. The mean number of ROH per individual (NROH) and per chromosome (NCROH), as well as the average length of ROH in Mbp per individual (LROH) and per chromosome (LCROH) were computed. Furthermore, the genomic inbreeding coefficient (FROH) was calculated by the total length of the genome comprised in the ROH for each individual divided by the total autosomal genome length (2,264,094,906 bp).

As used in several studies^9^^,^^10^ ROH_Islands were identified selecting the top 1% most frequent loci in the ROH, in order to detect more representative regions in this population.

Genes in ROH_Islands were annotated by Genome Data Viewer (https://www.ncbi.nlm.nih.gov/gdv/?org=sus-scrofa) using the ROH_islands coordinates.

Finally, the effective population size (Ne) in Cinghiato was also inferred using the SNePv1.1 software,^11^ which estimates historical Ne trend by analyzing the distribution of linkage disequilibrium (LD), directly derived from SNP genotypes, in relation to the recombination rate. The recombination rate between SNP pairs is estimated by SNePv1.1, based on the correlation between physical and genetic distances, with a default assumption of 1 Mb = 1 cM.

### Comparison with other Italian populations

To contextualize the Cinghiato population within a broader genetic landscape and to emphasize the genetic variability introduced through the reconstruction plan, a dataset was generated by merging the Cinghiato genomic data with those available online for other Italian breeds: Calabrese (n= 15), Casertana (n= 14), Cinta Senese (n= 13), Mora Romagnola (n= 9), Nero Siciliano (n= 15), Italian Wild Boars (n= 19) from the center of Italy and Sardinian Wild Boars (n= 20).^6^ The final dataset was consisted of 127 pigs with their genotypes at 34,510 autosome loci (resulted after a filtering based on a call-rate ≥ 95% and a MAF of 0.05). Comparing the Cinghiato population with these breeds allows for situating it within the genetic spectrum of Italian pig breeds, assessing its relatedness to both founder breeds (Cinta Senese and wild boars) and other autochthonous populations. This comparison also highlights the genetic impact of the reconstruction breeding process, contributing to a better understanding of the genetic variability underlying the Cinghiato’s distinct coat pattern.

The PCA and F_ST_ analyses via SVS software were performed to underline the genetic relationships among the Cinghiato and the other Italian breeds. PCA results were graphically visualized with the R package ggplot2.^7^ Finally, the ADMIXTURE analysis^12^ was performed identifying the most probable number of ancestral populations using the lowest cross-validation error (CV). The optimal number of clusters (K-value) in the ADMIXTURE analysis was set from 4 to 10.

## Cinghiato coat genes analysis

### *MC1R* and *KIT* genes: Sanger sequencing

All Cinghiato samples (plus one resulted with low quality in genotyping process) were sequenced to analyze the genetic makeup responsible for the variability of their belted coat pattern. This sampling also includes two pigs with a wild-colored coat, one of which has a white belt, in order to understand the genetic basis of the color of Cinghiato. The complete coding regions of the *MC1R* and *KIT* genes were sequenced (externally at BioFab Research) according to the protocol described by^13^ (all primers and annealing temperatures are reported in Table S1).

Polymorphisms were detected and visualized using GeneDoc 2.7 (http://nrbsc.org/gfx/genedoc) and Chromas 2.6.6 software (https://technelysium.com.au/wp/chromas/) respectively, with the AC141857 sequence included as a reference.

### Whole genome sequencing

**Figure 2.**
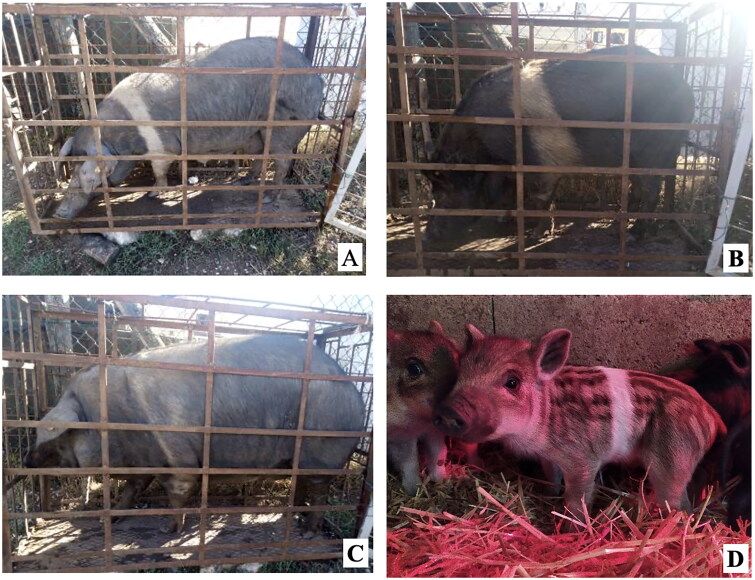
Coat color of whole genome sequenced samples. (A) White complete belt located at the shoulders, extended at forelimbs (SUP_18): this pattern reflects a broader expression of the desired belted phenotype, emphasizing complete belt coverage and extension beyond the shoulders. (B) White incomplete belt located over the shoulders; right forelimb is partially white left shoulder and forelimb not comprised (SUP_09): this incomplete symmetry demonstrates variability within the selection criteria and highlights challenges in achieving a uniform phenotype. (C) White complete thin belt located at shoulders, partially extended at right forelimb (SUP_16): the narrower belt reflects a phenotypic variation of the selected pattern, with partial forelimb involvement. (D) White belt located in the middle of the trunk, forelimbs and shoulders not comprised and wild boar coat color (SUP_19): this pattern aligns with historical depictions but contrasts with selection preferences targeting more consistent shoulder-centric belts as Cinta Senese.

A total of 4 pigs with extreme variation in coat color were chosen for WGS (Fig. 2) to analyze the variability in their genomes that could elucidate the genetic basis of their coat pattern. The variation in the belt pattern is observed in terms of its position along the trunk and symmetry: some belts are regular in width and symmetrical, while others are incomplete and asymmetrical, with different coverage on the left and right sides of the body.

The whole-genome sequencing was externally conducted by Novogene Corporation, which provided all the utilized procedures and pipelines as follows: i) library preparation was performed using the NEBNext^®^ UltraTM DNA Library Prep Kit for Illumina; ii) the qualified libraries were sequenced on Illumina platforms (Illumina NovaSeq 6000) using the 150 bp paired-end strategy. After sequencing, a quality control of reads was performed with Fastp software^14^ and the cleaned reads of 4 samples were aligned to the reference genome (GCF_000003025.6_Sscrofa11.1) using BWA software.^15^ SAMtools software was utilized to sort the .bam files and remove duplicates, as well as to detect and filter SNPs and InDels.^16^ Finally, the ANNOVAR software was used to annotate the detected variants.^17^

The sequences covering the MC1R and KIT genes have been extracted from each sample sequence (.bam files) using SAMtools package,^16^ in order to remap the positions of the mutations reported by^13^ according with the latest now available (GCF_000003025.6_Sscrofa11.1 assembly). In addition, having the entire genome of these four subjects available, the .bam files for other 8 genes involved in coat color variability (*ASIP, EDNRB, KITLG, MITF, OCA2, PAX3, SOX10, and TYRP1*) were extrapolated and analyzed.

## Results

### Genetic diversity indices and population structure of Cinghiato

**Figure 3.**
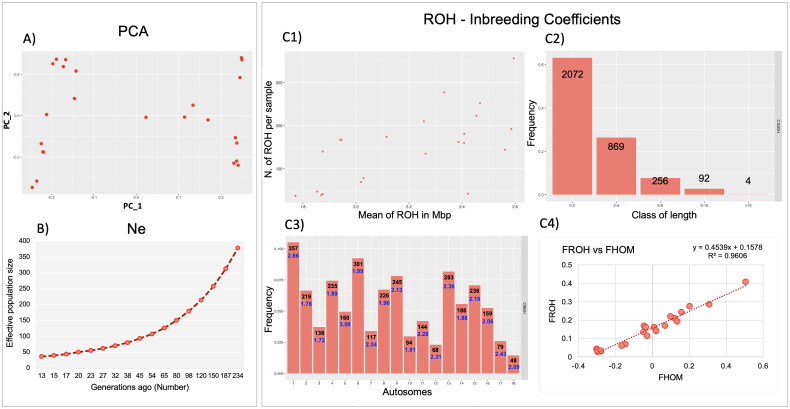
PCA and descriptive statistics calculated for Cinghiato population: (A) graphical representation of PCA: PC_1 (eigenvalues = 2.59; 29.41% of total genetic variability) vs. PC_2 (eigenvalues = 1.58; 13.61% of total genetic variability); (B) Effective population size (Ne); (C1) relationship between the number and mean length of ROH (Mbp = megabases); (C2) proportion of ROH for each class of length; (C3) proportion of ROH for autosome (black: n. of ROH; blue: mean ROH length); (C4) Linear regression and correlation values for F_HOM_ and F_ROH_.

The results of the genetic diversity indexes calculated in Cinghiato revealed a moderate level of observed and expected heterozygosity (H_O_ = 0.253 ± 0.206; H_E_ = 0.248 ± 0.186), with an average value of MAF of 0.185 ± 0.16. In the PCA plot (Fig. 3A), the population appears to separate along PC1 (i.e., the x-axis), suggesting the presence of detectable genomic variability among the analyzed Cinghiato pigs.

The lowest Ne values observed, ranging from 13 to 45 generations ago, are below 100 (Fig. 3B), which is commonly seen in small populations.^10^

### ROH-islands detection in Cinghiato

The analysis of runs of homozygosity pattern gave a total of 3,293 ROHs in the 22 individuals.

The mean NROH per sample was 149 ± 85.46, while the mean NCROH has a value of 207 ± 95.75. The 63% of the ROHs had length between 1 Mbp and 2 Mbp. ROHs between 2–4 and 4–8 Mbp were distributed with an incidence of 26% and 8% respectively, while long segments (>8 Mbp) represented only 2.9% of the total amount. The LROH was 2.29 ± 1.9 Mbp and the LCROH was 2.17 ± 0.35 Mbp (Fig. 3C1–C3).

The two genomic inbreeding coefficients showed negative values for F_HOM_ (−0.013 ± 0.211) and low positive value for the one calculated using ROH (F_ROH_ = 0.15 ± 0.098) (Fig. 3C4), and high correlation values(R2 = 0.96).

**Figure 4.**
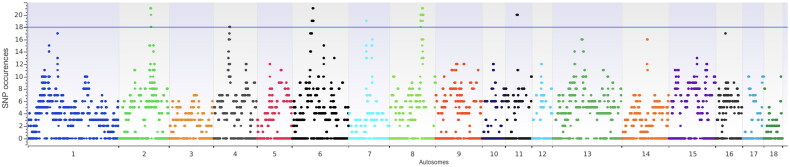
Manhattan plot of SNP incidences (y-axis) for Cinghiato population.

In Fig. 4, the Manhattan Plot shows the presence of ten ROH_Islands (identified as an island when present in at least 75% of the samples, Table 1) identified on six chromosomes

**Table 1.** ROH_Islands identified by selecting the top 1% most frequent loci in the ROH.

Chr	From	To	Length	nSNP	Genes
2	96,104,513	97,375,240	1,270,727	21	*MEF2C*
4	47,218,546	47,739,178		5	–
4	48,348,735	49,361,032		9	*MMP16*
6	60,024,687	63,470,629	3,445,942	41	*NLRP11, NLRP4, NLRP13, NLRP8, NLRP5, ZNF787, ZNF444, GALP, ZNF667, ZNF583, ZNF582, ZNF471, SMIM17, ZNF470, ZNF835, ZIM2, PEG3, DUXA, AURKC, ZNF304, ZNF416, ZNF134, ZSCAN4, ZNF671, C6H19orf18, ZNF135, ZNF329, ZNF8, A1BG, RPS5, RNF225, ZNF584, ZNF132, ZNF446, SLC27A5, ZBTB45, TRIM28, CHMP2A, UBE2M, MZF1, SAMD11, NOC2L, KLHL17, PLEKHN1, PERM1, HES4, ISG15, AGRN, RNF223*
7	52,177,010	52,709,566	532,556	15	*FSD2, MIR1839, AP3B2, CPEB1, RPS17, PDE8A, INSIG2, MIR9848, SLC28A1, ALPK3*
8	93,688,528	96,691,900	3,003,372	43	*C8H4orf33, JADE1, SCLT*
8	98,676,272	99,997,691	1,321,419	26	*FAT4, ANKRD50*
8	100,100,036	100,628,736	528,700	11	*SPRY1*
8	100,783,995	101,826,310	1,042,315	21	*SPRY1, SPATA5, NUDT6, FGF2, BBS12, IL21, IL2, ADAD1, KIAA1109*
11	34,119,552	36,493,548	2,373,996	24	*PCDH20*

Coordinate based on the Sscrofa11.1 genome assembly.

### Comparison with other Italian populations

**Figure 5.**
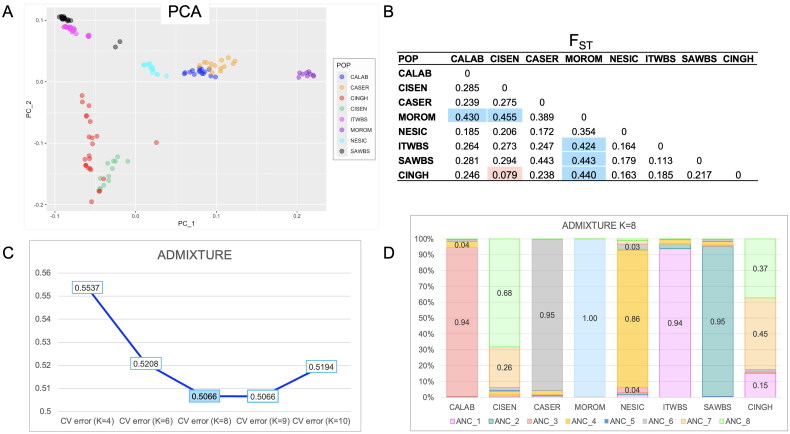
PCA analysis results. (A) Graphical representation of PCA: PC_1 (eigenvalues = 7.97; 21.1% of total genetic variability) vs. PC_2 (eigenvalue = 7.19; 19.04% of total genetic variability); (B) F_ST_ results: in pink the lowest value, in blue FST values > 0.4: (C) ADMIXTURE results: CV error values at different K (in blue the lowest at K = 8); (D) ADMIXTURE results: proportion of ancestors identified (‘ANC_n.’) at K = 8. CALAB (Calabrese); CISEN (Cinta Senese); CASER (Casertana); MOROM (Mora Romagnola); NESIC (Nero Siciliano); ITWBS (Italian wild boar); SAWBS (Sardinian Wild Boar); CINGH (Cinghiato).

The PCA performed on the merged dataset, which collectively accounts for 37.79% of the overall genetic variance, depicts the distribution of populations, with individuals grouping according to their breed. As expected, Cinghiato clustered together with Cinta Senese. Furthermore, Cinghiato (red dots) forms a vertically dispersed cluster (Fig. 5A).

The two populations of Wild Boar formed a distinct cluster, positioned along the axes of the Cinghiato-Cinta Senese cluster, underscoring a relative proximity between these populations. Notably, Casertana and Calabrese breeds exhibited a close clustering, whereas the Mora Romagnola breed defined its own distinct cluster. Additionally, the Nero Siciliano pigs grouped near the Calabrese breed, suggesting a connection between Casertana, Calabrese, and Wild boar populations.

The lowest, and most expected, value is the F_ST_ between Cinghiato and Cinta Senese (F_ST_ = 0.079) (Fig. 5B), given the utilization of the latter breed in the back breeding selection process, confirmes the genetic closeness. Additionally, relatively low levels of differentiation are evident between Cinghiato, Nero Siciliano, and the Italian Wild boar (F_ST_ = 0.163; 0.185). Moderate F_ST_ values are observed between Cinghiato and Calabrese, Casertano, and Sardinian Wild boar (F_ST_ = 0.246, 0.238, 0.217 respectively). The highest F_ST_ value of 0.440 detected between Cinghiato and Mora Romagnola pigs, indicates significant genetic differentiation between these populations. Similarly high F_ST_ values are observed between Cinta Senese and Mora Romagnola (F_ST_ = 0.455), as well as between Italian Wild boar, Sardinian Wild boar, and Mora Romagnola (F_ST_ = 0.424, 0.433). Additionally, also F_ST_ value between Mora Romagnola and Calabrese was > 0.4.

Regarding the ADMIXTURE analysis, the lowest CV error value (0.5066) was identified at K = 8 (highlighted in blue in Fig. 5C), indicating the optimal number of ancestral clusters for our dataset. Additionally, when focusing on the ADMIXTURE results at K = 8 (Fig. 5D), the proportion of identified ancestors (‘ANC_8’) aligns with the lowest CV error value.

## Cinghiato coat genes analysis

### *MC1R* and *KIT* genes: Sanger sequencing

As reported in Table 2, in *MC1R* only codon 122 resulted variable (G > A for SUP_9 and SUP_31 samples).

**Table 2.** MC1R polymorphisms at 7 codons.

Samples		Samples	Allele	Codons																				
				95			102			121			122			124			164			243		
**Sscrofa 11.1**	**Color coat**	**Color coat**	**MC1R (E^+^)**	**G**	**T**	**G**	**C**	**T**	**G**	**A**	**A**	**T**	**G**	**T**	**C**	**G**	**A**	**C**	**G**	**T**	**G**	**G**	**C**	**A**
SUP_01	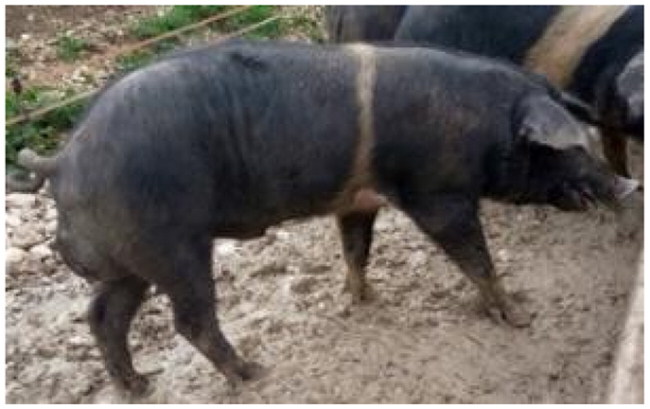	White complete thin belt located over the shoulders, forelimbs not comprised.	MC1R (E^D2^)	.	.	.	.	.	.	.	.	.	.	.	.	A	.	.	.	C	.	.	.	G
SUP_02	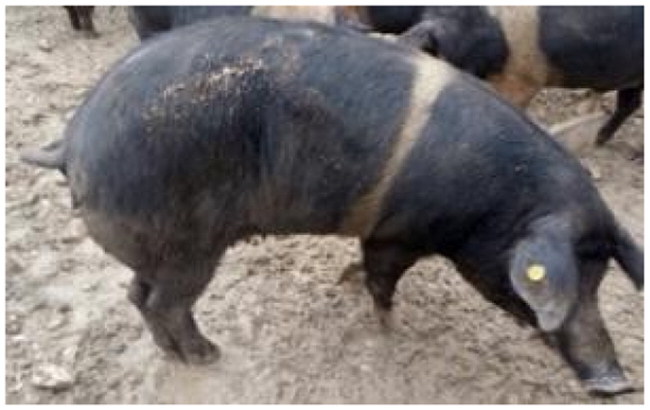	White complete thin belt located over the shoulders, right forelimb is partially white.	MC1R (E^D2^)	.	.	.	.	.	.	.	.	.	.	.	.	A	.	.	.	C	.	.	.	G
SUP_03	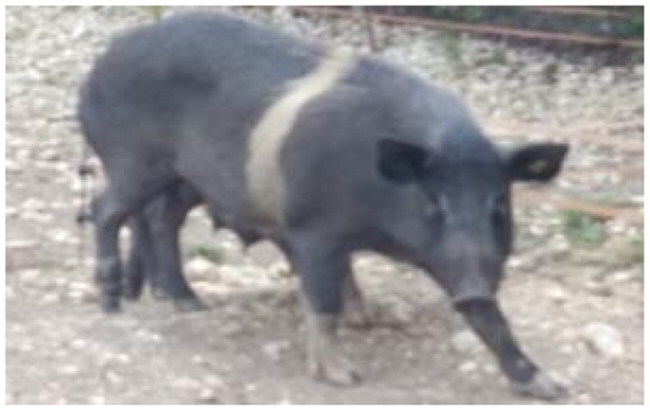	White incomplete belt (only presents on right side) located in the middle of the trunk, right forelimb is partially white.	MC1R (E^D2^)	.	.	.	.	.	.	.	.	.	.	.	.	A	.	.	.	C	.	.	.	G
SUP_04	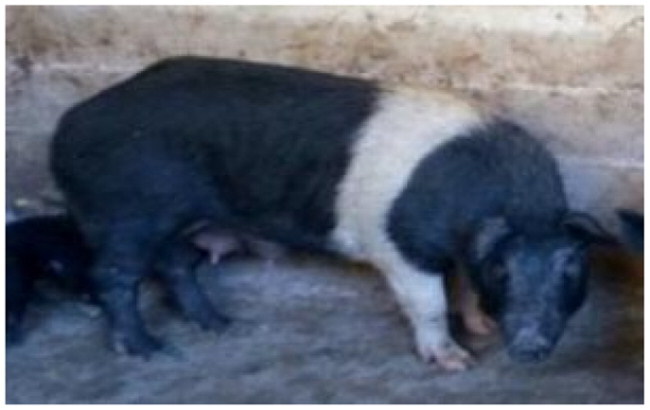	White complete belt located at shoulders, extended at forelimbs.	MC1R (E^D2^)	.	.	.	.	.	.	.	.	.	.	.	.	A	.	.	.	C	.	.	.	G
SUP_06	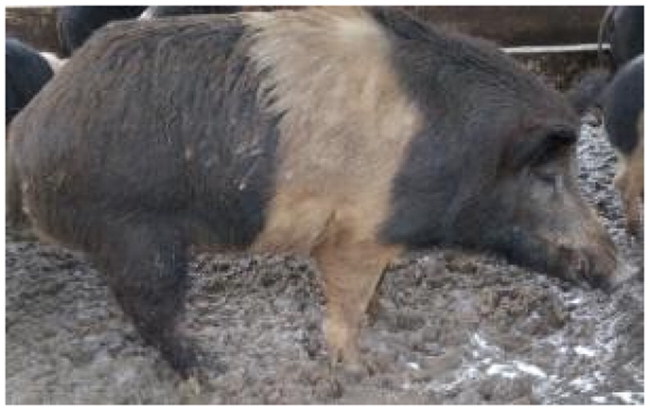	White complete belt located at shoulders, extended at forelimbs to middle of the trunk. Wild boar color coat.	MC1R (E^D2^)	.	.	.	.	.	.	.	.	.	.	.	.	A	.	.	.	C	.	.	.	G
SUP_08	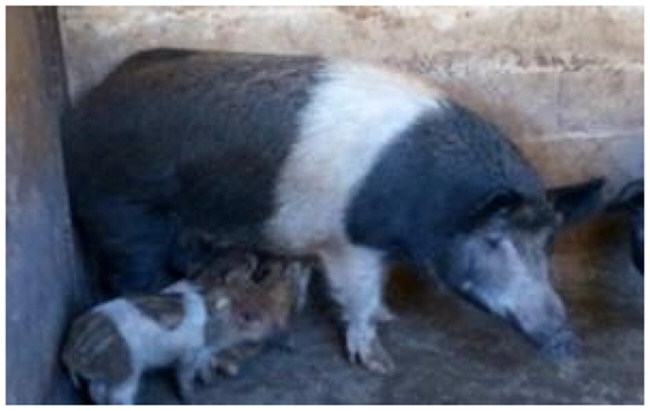	White complete belt located at shoulders, extended at forelimbs to middle of the trunk. Wild boar color coat.	MC1R (E^D2^)	.	.	.	.	.	.	.	.	.	.	.	.	A	.	.	.	C	.	.	.	G
**SUP_09**	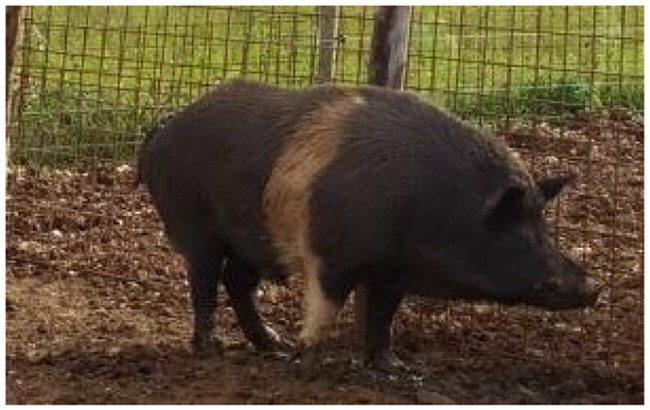	White incomplete belt located over the shoulders, right forelimb is partially white. Left shoulder and forelimb not comprised.	MC1R (E^D2^)	.	.	.	.	.	.	.	.	.	.	.	.	A	.	.	.	C	.	.	.	G
SUP_10	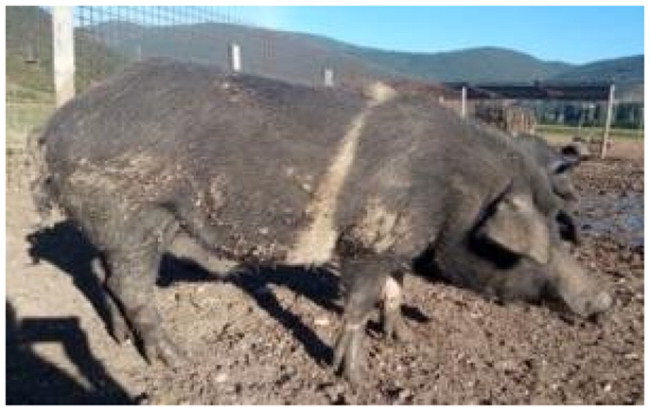	White complete thin belt located over the shoulders, forelimbs are partially white.	MC1R (E^D2^)	.	.	.	.	.	.	.	.	.	.	.	.	A	.	.	.	C	.	.	.	G
SUP_11	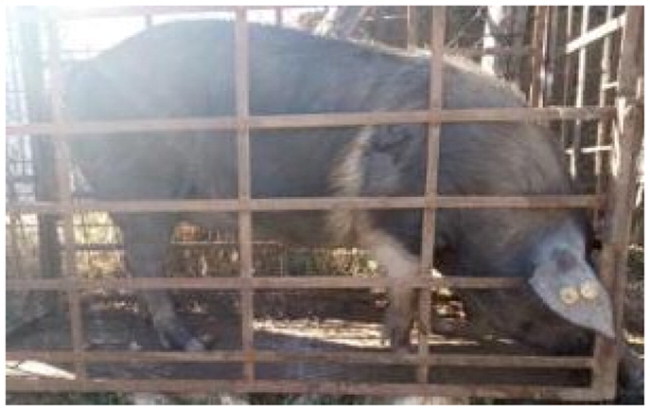	White incomplete thin belt located over the shoulders, right shoulder and forelimb is partial white, left forelimb is not comprised.	MC1R (E^D2^)	.	.	.	.	.	.	.	.	.	.	.	.	A	.	.	.	C	.	.	.	G
SUP_12	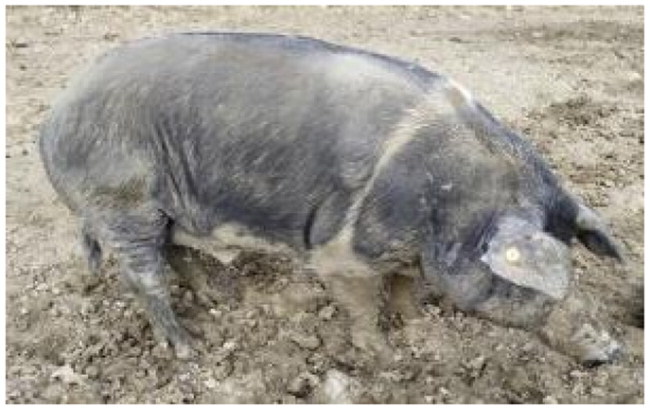	White complete thin belt located across the shoulders, shoulders and forelimbs are white.	MC1R (E^D2^)	.	.	.	.	.	.	.	.	.	.	.	.	A	.	.	.	C	.	.	.	G
SUP_13	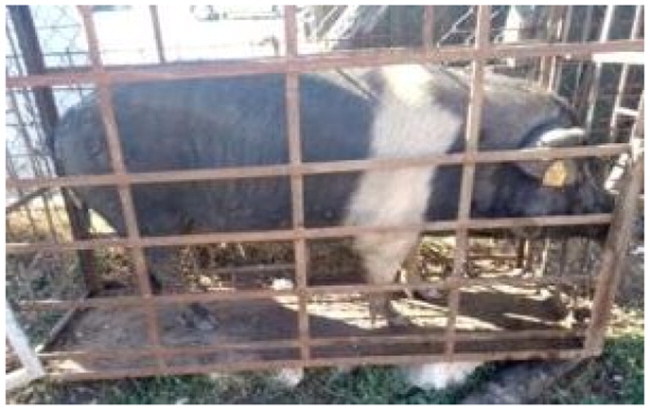	White complete belt located at shoulders, extended at forelimbs.	MC1R (E^D2^)	.	.	.	.	.	.	.	.	.	.	.	.	A	.	.	.	C	.	.	.	G
SUP_14	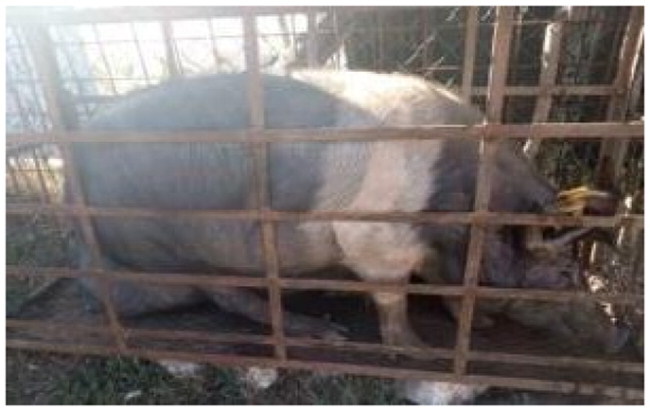	White complete belt located at shoulders, extended at forelimbs.	MC1R (E^D2^)	.	.	.	.	.	.	.	.	.	.	.	.	A	.	.	.	C	.	.	.	G
SUP_16	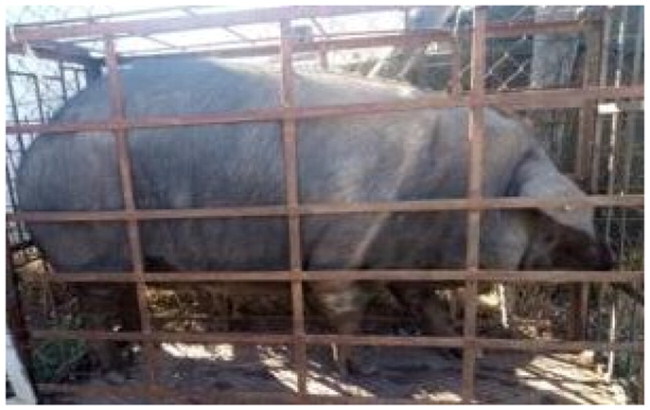	White complete thin belt located at shoulders, partially extended at right forelimb.	MC1R (E^D2^)	.	.	.	.	.	.	.	.	.	.	.	.	A	.	.	.	C	.	.	.	G
SUP_17	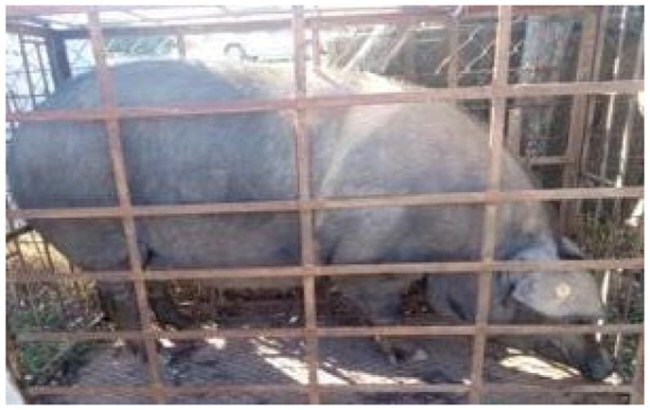	White incomplete thin belt located over the shoulders, forelimbs not comprised.	MC1R (E^D2^)	.	.	.	.	.	.	.	.	.	.	.	.	A	.	.	.	C	.	.	.	G
SUP_18	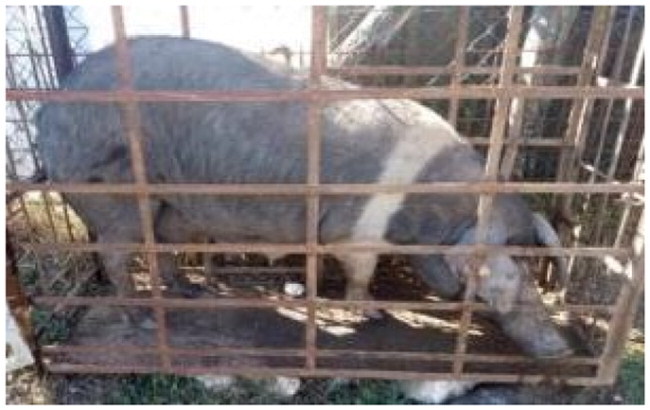	White complete belt located at shoulders, extended at forelimbs.	MC1R (E^D2^)	.	.	.	.	.	.	.	.	.	.	.	.	A	.	.	.	C	.	.	.	G
SUP_19	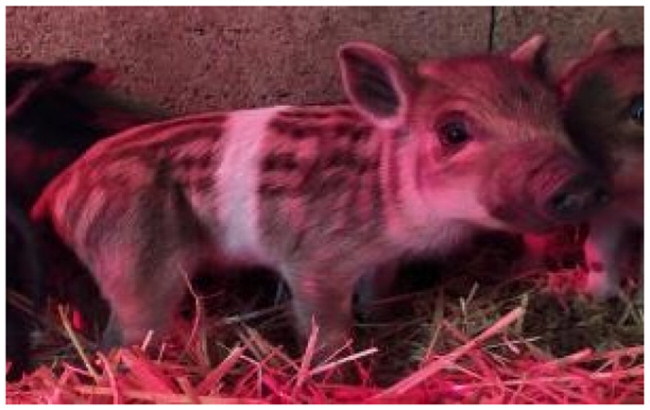	White belt located in the middle of the trunk, forelimbs and shoulders not comprised. Wild boar color coat.	MC1R (E^+^)	.	.	.	.	.	.	.	.	.	.	.	.	**G**	.	.	.	C	.	.	.	G
SUP_20	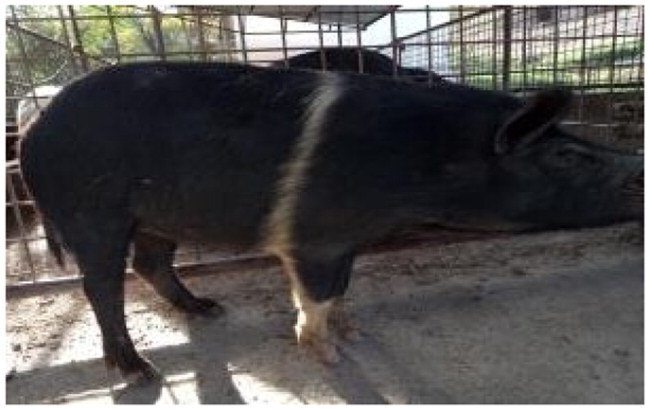	White complete thin belt located over the shoulders, forelimbs partially white.	MC1R (E^D2^)	.	.	.	.	.	.	.	.	.	.	.	.	A	.	.	.	C	.	.	.	G
SUP_21	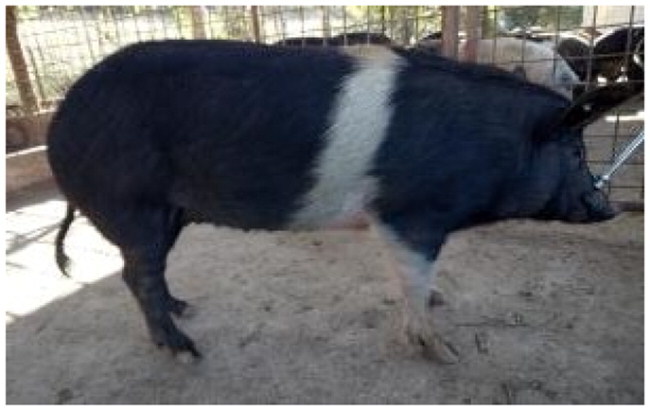	White complete belt located over the shoulders, right forelimb is partially white, left forelimb not comprised.	MC1R (E^D2^)	.	.	.	.	.	.	.	.	.	.	.	.	A	.	.	.	C	.	.	.	G
SUP_22	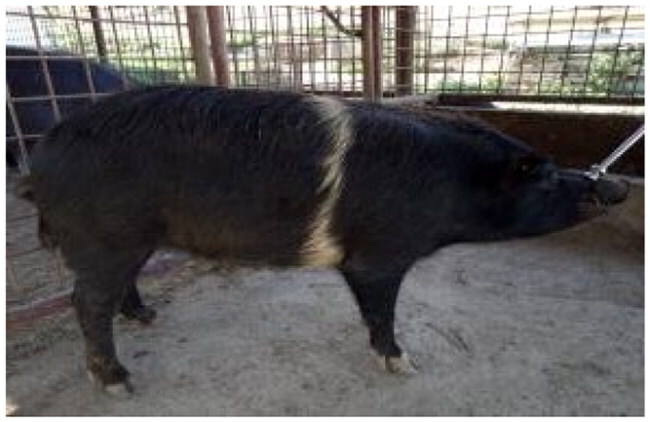	White complete thin belt located over the shoulders, forelimbs not comprised.	MC1R (E^D2^)	.	.	.	.	.	.	.	.	.	.	.	.	A	.	.	.	C	.	.	.	G
SUP_23	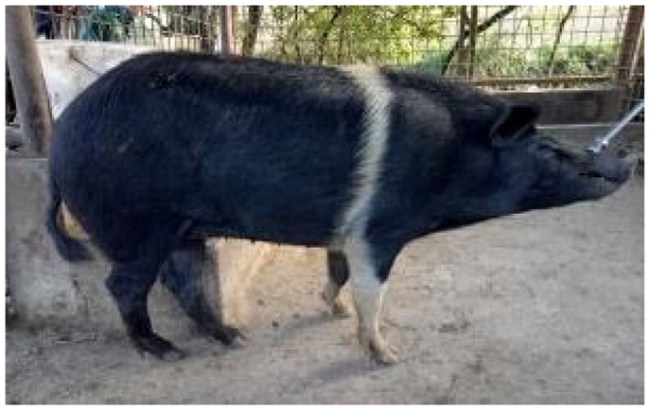	White complete thin belt located over the shoulders, forelimbs partially white.	MC1R (E^D2^)	.	.	.	.	.	.	.	.	.	.	.	.	A	.	.	.	C	.	.	.	G
SUP_24	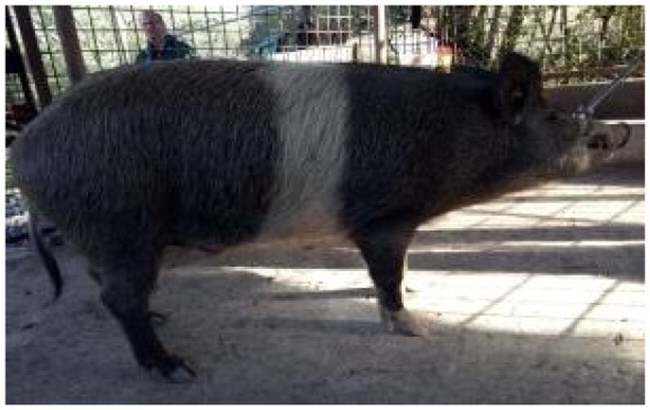	White complete belt located over the shoulders, forelimbs are not comprised. Wild boar color coat.	MC1R (E^D2^)	.	.	.	.	.	.	.	.	.	.	.	.	A	.	.	.	C	.	.	.	G
SUP_30	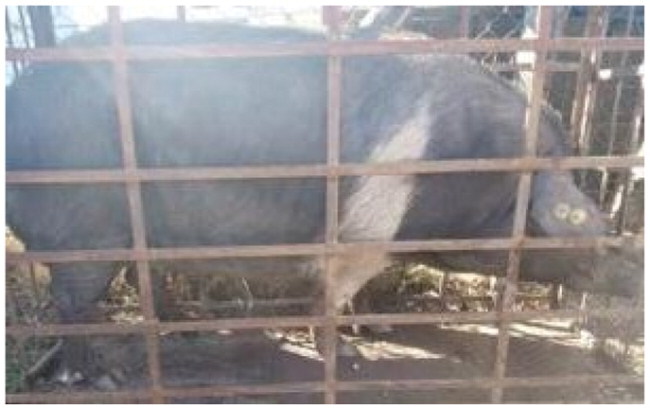	White incomplete belt located at shoulders, extended at right forelimb, left forelimb not comprised.	MC1R (E^D2^)	.	.	.	.	.	.	.	.	.	.	.	.	A	.	.	.	C	.	.	.	G
SUP_31	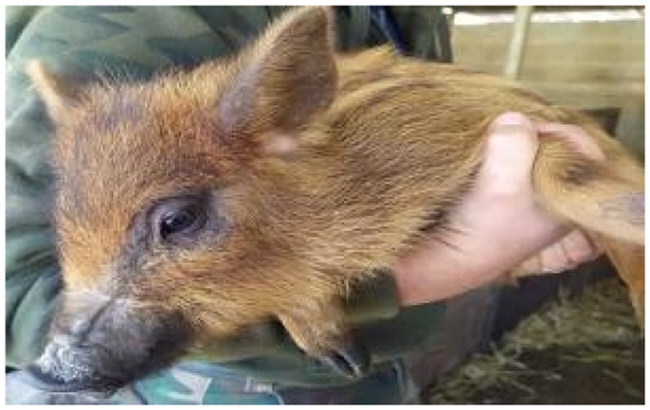	No Belt. Wild boar color coat	MC1R (E^+^)	.	.	.	.	.	.	.	.	.	.	.	.	**G**	.	.	.	C	.	.	.	G

This polymorphism (G) at the first nucleotide of codon 122 corresponds to the wild type allele (E^+^), instead all other samples resulted E^D2^ (all A). Details regarding the *MC1R* and *KIT* exons positions (according to the Sscrofa11.1 assembly) are summarized in Table S1.

Table S2 reports all polymorphisms (n. 69) found for *KIT* gene: mutations confirmed by whole-genome sequencing (WGS) and Sanger sequencing are highlighted in red. At the positions 41,445,577 (C > T) and 41,445,580 (G > A) located in exon 3 (corresponding to g.58361C > T and g.58364G > A in^13^) the polymorphism observed (homozygous vs. heterozygous genotypes) did not match the expected association with *KIT* duplication and the belt phenotype described by.^13^ Specifically: n. 17 belted samples resulted homozygous at these loci, n=6 belted samples (including sample SUP_31 with wild board phenotype, no belt, Table 2), were heterozygous at both loci (Fig. S1).

### Whole genome sequencing

**Figure 6.**
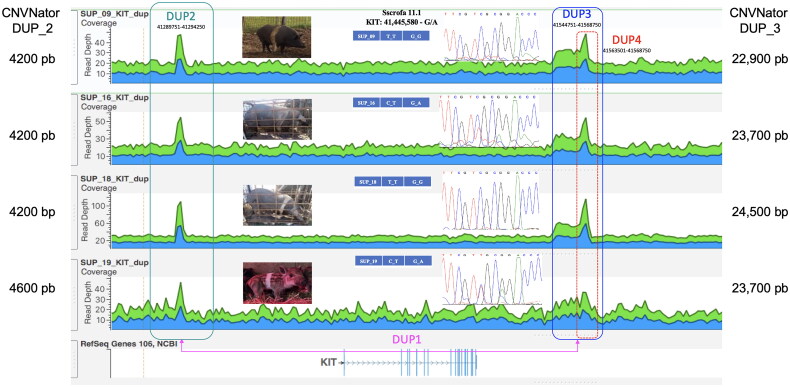
WGS results for four Cinghiato pigs. Indications of duplications involving *KIT* gene were highlighted in colored box. Length of CNV duplications identified by CNVnator software was also provided.

No new mutations were identified analyzing the *MC1R* gene. The Dominant white/*KIT* locus exhibits genetic instability. The allele officially linked to the belted phenotype is I^Be^ (also denoted as I^Be1^), characterized by a single copy of the *KIT* gene, one DUP1 region, and multiple copies of DUP2, DUP3, and DUP4. As reported in Fig. 6, all belted pigs were I^Be^. DUP2 and DUP3 duplications were also detected by CNVnator software utilizing the default parameters.^18^ Details of duplicate lengths are reported in Fig. 6.

Tables 3 and 4 reported the polymorphisms found for the eight genes analyzed in the four Cinghiato samples. For each gene its GeneBank number (NCBI online database) considered was reported. All identified mutations were verified by consulting the list of variants catalogued in the ‘variant table’, available for the annotated gene in Ensembl database as at https://www.ensembl.org/Sus_scrofa/Gene/Variation_Gene/Table?db=core;g=ENSSSCG00000005193;r=1:209725638-209745705;t=ENSSSCT00000005725.

**Table 3.** Polymorphisms at *SOX10, EDNRB, OCA2*, and *PAX3* genes with respect to the Sscrofa 11.1 reference (Duroc breed).

Samples		^1^*SOX10*; Chr5: 9,890,439 - 9,901,746			^2^*EDNRB*; Chr11: 50,075,782 - 50,101,836	^3^*OCA2*; Chr15: 56,657,648 - 56,869,920	^4^*PAX3*; Chr15: 124,093,803 - 124,193,041		
		EX 3 9,892,425	9,900,885 3UTR variant	9,900,998 3UTR variant		5UTR 56,657,666	EX 2 124,191,176 synonymous variant 189C > T→Ala63=	EX 8 124,095,914 Missense variant 1334C > G →Ser445Cys	124,095,172 3UTR variant
Sscrofa 11.1	Coat color	CGG/GGG (Fig. S2A)	A/A	C/C		C/C	G/G	G/G	C/C
SUP_09	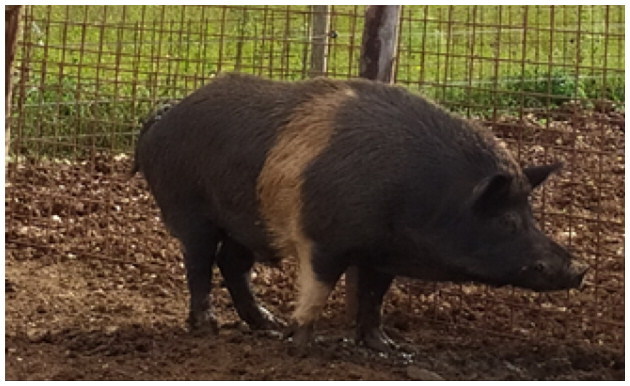	CGG/DEL	A/C	C/T	No mutation in every exon comparing with reference	C/C	A/A	G/C	T/T
SUP_16	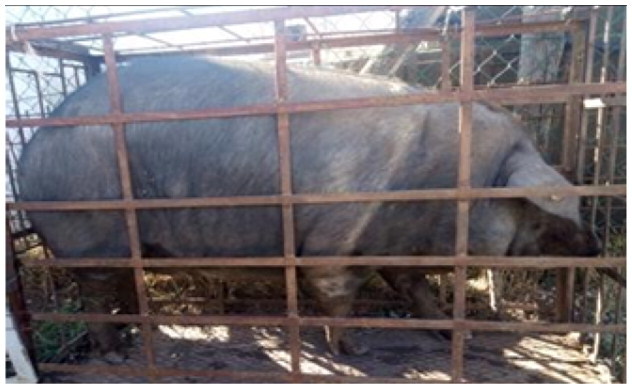	DEL/DEL	C/C	T/T	No mutation in every exon comparing with reference	C/C	G/G	G/C	T/T
SUP_18	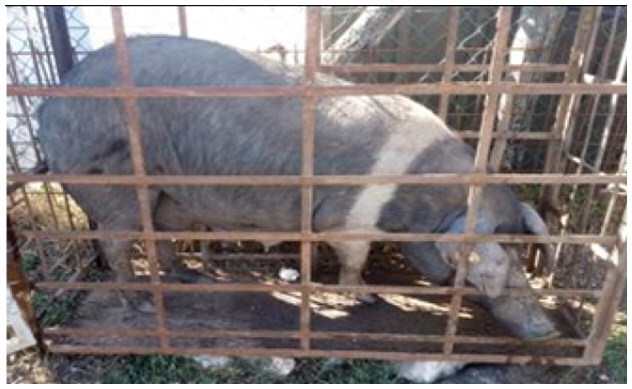	DEL/DEL	C/C	T/T	No mutation in every exon comparing with reference	C/C	G/G	C/C (Fig. S2B)	T/T
SUP_19	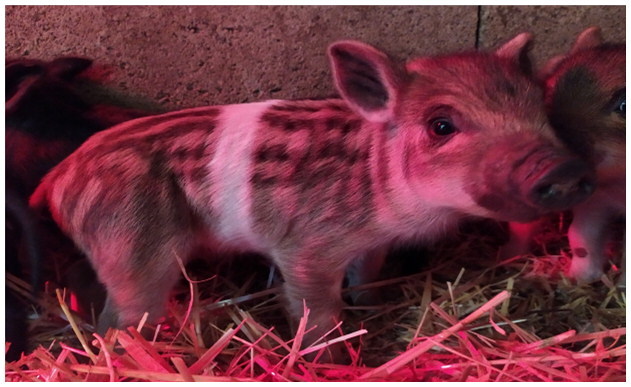	DEL/DEL	A/C	C/T	No mutation in every exon comparing with reference	G/G	G/G	G/G	T/T

**Table 4.** Polymorphism at *TYRP1, MITF, ASIP*, and *KITLG* genes, respect to Sscrofa 11.1 reference (Duroc breed).

Samples		^5^*TYRP1*; Chr1: 209,726,606 - 209,744,584	^6^*MITF*; Chr13: 51,177,356–51,422,09	^7^*ASIP*; Chr17: 37,542,615–37,718,787	^8^*KITLG*; Chr5: 94,016,992 - 94,110,219
		EX 2 209,742,958 Missense variant 503A > G → His168Arg	Intron* 51,402,263 - 51,404,538	Intron 37,645,842 − 37,646,391	EX 8 94,096,799 Missense variant 658A > G →Thr220Ala
Sscrofa 11.1	Coat color	T/T	-/- (Fig. S2D)	-/- (Fig. S2E)	A/A
SUP_09	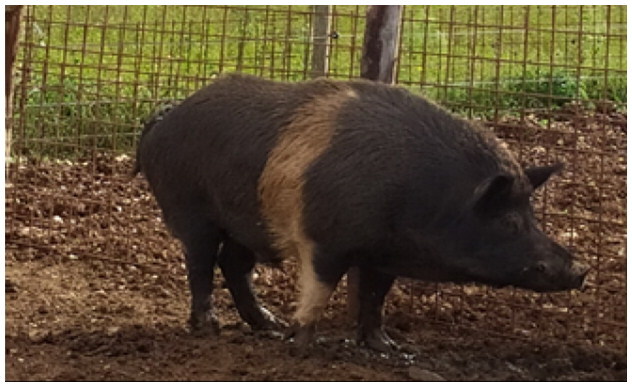	T/C	-/Deletion of about 1000 bp	Homozigote Duplication of about 500 bp	**G/G** (Fig. S2E)
SUP_16	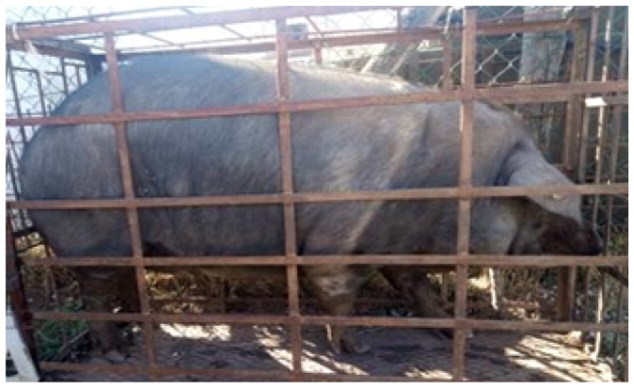	T/T (based on read depth ratio of the two bases) (Fig. S2C)	Homozygote Deletion of about 1000 bp	Homozygote Duplication of about 500 bp	**G/G** (Fig. S2F)
SUP_18	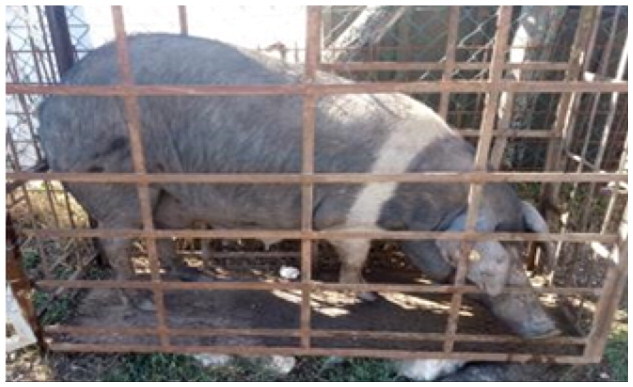	**C/C** (Fig. S2C)	Homozygote Deletion of about 1000 bp	Homozygote Duplication of about 500 bp	A/A
SUP_19	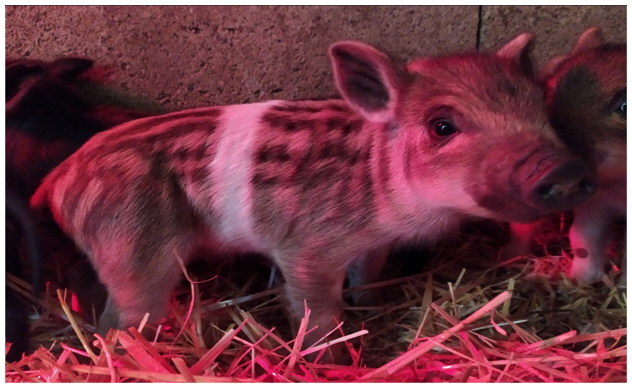	T/C	Homozygote Deletion of about 1000 bp	Homozygote e Duplication of about 500 bp	**G/G** (Fig. S2F)

## Discussion

### Genetic diversity indexes and population structure of Cinghiato

Genetic diversity remains significant even in the context of a reconstruction breeding plan, serving as the foundation for sustainable breeding programs and ensuring their success thorough time. The Cinghiato population, despite its small size, demonstrates moderate genetic diversity, which aligns with expectations for a breeding program aimed at its reintroduction into the farming system as a local breed. The reconstruction project was initiated by the farmers themselves and supported by institutions that, recognizing the importance and uniqueness of this breed, endorsed the effort to recover the Cinghiato pig.^5^

The observed heterozygosity (H_O_) and expected heterozygosity (H_E_) in Cinghiato indicate a moderate level of genetic diversity. Comparative analyses revealed that genetic diversity in Cinghiato is slightly lower than in other European local pig breeds, such as Cinta Senese (H_O_ = 0.300; H_E_ = 0.300) and Casertana (H_O_ = 0.291; H_E_ = 0.327).^19^ However, these values align closely with other small local pig populations, such as Nero Siciliano and Calabrese, which showed comparable H_O_ and H_E_ values (H_O_ and H_E_ respectively: 0.309–0.325; 0.296–0.255).^20^ The observed values in Cinghiato also align with those reported for Italian Wild Boar (H_O_ = 0.197; H_E_ = 0.212) and Cinta Senese (H_O_ = 0.212; H_E_ = 0.212) in the same study.^20^

While slightly lower than the average for European local pig breeds (H_O_ = 0.297; H_E_ = 0.303), these findings reflect the population’s history as a young, small population derived from crossbreeding and focused on phenotypic traits.

Regarding the F_HOM_ result, Cinghiato showed a negative value indicating a low inbreeding level. This result is in line with those observed in another article,^12^ in Cinta Senese (F_HOM_ = −0.034), and Italian Landrace (F_HOM_ = 0.003).^21^

Accordingly, values of F_ROH_ observed in Cinghiato are consistent with those observed in Nero Siciliano (F_ROH_ = 0.10), but lower than those identified in Casertana, Cinta Senese, Mora Romagnola (F_ROH_ = 0.23; 0.27; 0.46), confirming a low level of inbreeding.^20^

These findings suggest several considerations. First, efforts to plan mating in Cinghiato to avoid inbreeding have successfully maintained a low level of inbreeding within the population. However, it is important to note that while negative F_HOM_ values may suggest random mating among individuals, the small sample size and random sampling errors could also lead to a negative result.^22^

The observed MAF value in Cinghiato is lower compared to Nero Siciliano, Calabrese, and Casertana (MAF = 0.241, 0.194; 0.217) and higher than that of Cinta Senese and Italian Wild Boar (MAF = 0.156; 0.158);^20^ Similar higher values were observed in Nero Siciliano, Casertana, Cinta Senese, and Wild Boar in other studies (MAF = 0.272; 0.246; 0.220; 0.192).^19^

Overall, SNP analysis provides a moderate level of informativeness across the majority of examined breeds, as these local breeds are not the primary focus for the development of commercial porcine chips.^19^^,^^23^

The estimation of effective population size (Ne) provides valuable insights into the level of genetic diversity within a population, particularly in autochthonous breeds and breeding nuclei. Findings regarding Ne align with those observed in similar populations, highlighting the genetic constraints faced by these breeds. The lowest Ne values observed, particularly for the 13th generation presumed to be the contemporary Ne, fall below the threshold of 100. It should be emphasized that an effective population size of 100 is recommended to maintain the genetic diversity of a population.^24^ Muñoz et al. 2019 observed very low values of Ne, particularly, in breeds such as Casertana, Apulo Calabrese, Turopolje, Mora Romagnola and in two Lithuanian pig breeds, confirming a strong need to implement conservation strategies.^19^

### ROH_Islands detection in Cinghiato

The method based on ROH is a powerful approach for estimating genomic inbreeding, especially in local livestock populations.^25^^,^^26^ Long ROH segments are usually associated with recent inbreeding events due to their sensitivity to population changes.^26^

In Cinghiato, the results of LROH and LCROH, mostly under 3 Mb (with over 60% below 2 Mb), suggest that inbreeding occurred not recently, but likely before the reconstruction project. Despite this, the number of ROHs (3293) indicates a long period of small population size, though the source population was likely substantial.^26^^,^^27^

Out of 18 autosomes analyzed, ROH islands were identified in ten. Some of these regions are associated with traits in other pig populations.

- *SSC2*: The *MEF2C* gene, annotated in this ROH island, is linked to meat quality and muscle glycogen storage in various pig breeds,^28^^,^^29^ and reproductive traits in commercial pigs.^29^
- *SSC4*: Two ROH islands here overlap with those identified in Polish commercial pig breeds.^30^ *MMP16*, located in the second island, is involved in cartilage formation, regeneration, and neurological disorders in Chinese pigs.^31^
- *SSC6*: A ROH island covers QTL regions related to leg quality (front and hind) and overall conformation in Danish pig breeds.^32^ This region contains several *ZNF* genes involved in various biological processes, such as DNA recognition, transcriptional activation, protein folding and assembly, and lipid binding.^33–35^ Specific ZNF genes like *ZNF583, ZNF471, ZNF677*, and *ZNF444* are also implicated in diverse roles from muscle weakness and fertility in pigs^36–38^ to cell growth and apoptosis.^33^^,^^35^ Additionally, *NLR* family members (e.g., *NLRP11, NLRP13*) are linked to immune-inflammatory responses.^39^^,^^40^ Other genes like GALP and TRIM28 are important for appetite regulation, inflammation, energy metabolism, and immune responses.^41–43^ *SAMD11* significantly influences meat quality by affecting meat-to-fat ratios and intramuscular fat deposition, as well as *SLC27A5* in Gochu Asturcelta pig.^44^^,^^45^ Lastly, PEG3 plays significant roles in physiological and developmental functions and is also involved in embryonic and placental development.^41^^,^^46^
- *SSC7*: The *FSD2* gene, found in the ROH island on *SSC7*, influences meat quality, affecting intramuscular fat content and color.^47^ The *CPEB1* gene is involved in reproductive processes in pigs, impacting fertility and meiotic resumption.^22^ *INSIG2* plays a key role in cholesterol metabolism and fetal development.^48^
- *SSC8*: The ROH island identified on SSC8 overlaps with QTLs for subcutaneous fat thickness in lean-selected boar lines^49^ and body length (*JADE1* gene) in crossbred pigs.^50^ It also coincides with three selective sweep signals in Chinese pig breeds adapted to altitude^51^ and has been detected in other Italian and European local breeds.^19^^,^^20^ Genes within this region, such as *KIAA1109,*^52^ *NUDT6, SPATA5, FGF2,*^53^ and *SPRY1*, are associated with traits like feed efficiency, obesity, fat deposition, immunity (*IL2*), and male fertility (*ADAD1*).^54^ Notably, SPRY1 plays a complex role in fat regulation in mice,^55^ and may influence lean meat content and thermal adaptation in pigs.^48^
- *SSC11*: The ROH island on SSC11 overlaps with one identified in the Nero Siciliano breed.^20^ The *PCDH20* gene, although not associated with any major economic traits, is linked to brain activity and tameness in indigenous pig breeds.^56^

The identification of ROH islands in the Cinghiato population highlights genomic regions that could be relevant for future breeding plans. These regions may harbor genes linked to important traits like meat quality, reproductive efficiency, and fat deposition. While no definitive conclusions can be made regarding Cinghiato pigs, further studies with larger populations will help to better understand these associations and their potential applications in breeding.

### Comparison with other Italian populations

As expected, Cinghiato shows strong genetic affinity with Cinta Senese—the breed used in its development—as demonstrated by PCA, FST, and ADMIXTURE analyses. The PCA plot reveals clear breed-based clustering, with Cinghiato closely grouped with Cinta Senese, though its sparse cluster indicates notable genetic and phenotypic variability (Table 2).

The positioning of two Wild Boar populations near the Cinghiato–Cinta Senese cluster reflects genetic introgression from planned crossings. In Cinta Senese, this proximity may also result from outdoor breeding systems that increase contact with wild boars.^57^

Gene flow between wild boar and local breeds is further suggested by the clustering of Casertana and Calabrese with wild boars, while Nero Siciliano acts as a genetic bridge, showing close affinity supported by the presence of the wild-type *E^+^ MC1R* allele.^20^^,^^58^ Mora Romagnola forms a distinct cluster, consistent with prior studies showing its genetic separation from other breeds and lack of affinity with Italian Wild Boar.^19^^,^^20^^,^^53^^,^^59^

F_ST_ and ADMIXTURE analyses align with previous findings on genetic diversity, indicating that most variation occurs within rather than between local breeds.^60^ The observed F_ST_ values are in line with European data (avg. 0.134, range 0.021–0.209)^19^^,^^60^ and with studies involving 11 European breeds (F_ST_ = 0.27), confirming that differentiation among Cinghiato, Casertana, and Calabrese mirrors trends seen across Europe.^60^^,^^61^ The lowest F_ST_ between Cinghiato and Cinta Senese confirms their close genetic relationship, while moderate values with Wild Boar reflect hybrid use in reconstruction. The highest F_ST_ between Cinghiato and Mora Romagnola highlights significant divergence, in line with the known closeness of Mora Romagnola to Duroc.^53^^,^^58^^,^^59^^,^^62^ ADMIXTURE results reinforce the Cinghiato–Cinta Senese link. Notably, it’s intriguing that there is a portion of unknown ancestry in common between Cinghiato and Cinta Senese that cannot be attributed to the genetic introgression of the other autochthonous breeds analyzed in the study. Additionally, contributions from Wild Boar confirm introgression through the reconstruction plan. Calabrese^59^ and Mora Romagnola,^20^ maintain distinct genetic identities, as supported by previous research.

## Cinghiato coat genes analysis

### *MC1R* and *KIT* genes: Sanger sequencing

*MC1R* gene polymorphisms analysis has shown that only two samples (SUP_9 and SUP_31), exhibited a variation at codon 122 (G > A), revealing the presence of the wild type allele (E^+^), while all other samples consistently showed the E^D2^ allele (A). These findings align with expectations, as the E^D2^ allele is prevalent in numerous European breeds known for solid black patterns, such as Hampshire, Basque, Gascon, Schwäbisch-Hällisches, and including Cinta Senese.^63^

Notably, only the SUP_9 and SUP_31 samples deviated by showing the wild type allele (E^+^), while displaying coat striping and a reddish color like that of wild boars. This is consistent with the allele being considered ‘wild’ and predominantly found in wild boar populations known for their reddish coat color and also observed in Mangalitza and Mora Romagnola.^63^^,^^64^

The examination of *KIT* polymorphisms highlights an intriguing aspect of the belted phenotype genetics. The presence of polymorphisms at positions 41,445,577 (C > T) and 41,445,580 (G > A) in exon 3 of the *KIT* gene, as reported in the present study, does not correlate with the known mechanism associated with the belted phenotype dictated by allele I^Be1^.^13^

This allele, characterized by a single copy of the *KIT* gene producing a complete functional protein without the splice mutation, typically manifests with multiple copies of the DUP2, DUP3, and DUP4 regions, which have been associated to distinctive white belt encompassing the shoulders and forelegs seen in breeds like Cinta Senese, Angler Sattelschwein, British Saddleback, and Hampshire.^53^^,^^63^^,^^65–67^ However, our findings show that among the belted samples, 17 were homozygous and 6 were heterozygous (including sample SUP_31 with a wild boar phenotype and no belt), suggesting a possible deviation or additional complexity in the genetic basis of the belt phenotype in this population.

This could indicate variations similar to those seen in other breeds like Krskopolje and Schwäbisch-Hällisches Schwein, showing variation in belt in terms of width, where another allele (I^Be2^) noted for a single DUP1 region but varied copies of DUP2 and DUP4 and only one of DUP3 might be influencing the phenotypes.^68^

The examination of *KIT* and *MC1R* polymorphisms alone fails to offer a distinct understanding of the phenotypical variations noted in the Cinghiato population. Notably, the genetic composition at these two genes is quite uniform (with some exception for two allelic forms of *MC1R*) across the four samples, implying minimal impact on the size and positioning of the belt.

### Whole genome sequencing

In our whole genome sequencing analysis, numerous polymorphisms were identified across various genes; however, no new mutations were detected in the *KIT* and *MC1R* genes.

Tables 3 and 4 detail the polymorphisms observed in the eight analyzed genes across the four Cinghiato samples. While some of these polymorphisms have been previously associated with coat color variation, they do not explain the specific coat patterns observed in the Cinghiato samples.

The *SOX10* gene, known for its role in melanocyte development, has been implicated in coat pattern variations, as seen in Chinese Bama miniature pigs, which display a distinctive two-end black pattern with a broad central white area due to melanocyte deficiency.^69^ However, the mutations in *SOX10* identified in the Cinghiato samples cannot be linked to any known phenotypic differences, as the spontaneous nature of these mutations often complicates genotype-phenotype correlations.^70^^,^^71^

The influence of the *OCA2* gene on pigmentation remains unclear, though its association with a specific red-colored strain of Iberian pigs has been characterized by the segregation of three SNPs forming two intragenic haplotypes.^72^ Notably, a polymorphism at *OCA2* was identified in a sample with a reddish, wild boar-like coat pattern (SUP19), validating its potential role in coat color variation. This finding underscores the need for further investigation into *OCA2* and its interactions with *MC1R*, which may enhance our understanding of pigmentation genetics in pigs.^72^ The genes *EDNRB* and *TYRP1*, both linked to coat color variation in Asian pig lineages, revealed contrasting results. No polymorphisms were detected in *EDNRB*, the candidate gene for the two-end black coat pattern in Chinese pig breeds.^73^ Conversely, polymorphisms were identified in the *TYRP1* gene, typically associated with the Brown locus,^63^ but they did not correlate with phenotypic variations in European pigs or our samples.

A polymorphism was also identified in the *ASIP* gene; however, its role in phenotype variation, particularly in dorsoventral coloration,^74^ remains inconclusive. Previous studies suggest that coat color polymorphisms and *MC1R/ASIP* mutations are prevalent in wild and domestic hybrids, indicating a possible synergistic effect between these genes.^74^ Further investigation into their interaction could be crucial for a deeper understanding of their collective influence on pigmentation, particularly in hybrids. Additionally, polymorphisms were found in the *MITF* and *TYRP*2 genes, both known to contribute to pig coat pigmentation.^63^ *MITF*, in particular, has been associated with the white-spotted patterns observed in certain Chinese pig breeds, such as Tongcheng pigs and Chinese wild boars, although the specific regulatory mutations remain undefined.^75^ Investigating the potential interaction between *MITF* and *TYRP2* could provide valuable insights into pigmentation mechanisms.

Lastly, the role of *KITLG* in coat pigmentation is well acknowledged, although the functional significance of the observed polymorphisms and selection signatures in various pig breeds, including our samples, requires further clarification. Overall, these findings highlight the need for additional research to unravel the genetic mechanisms underlying belt pattern variation and position.

## Conclusion

This study aimed to explore the genetic basis underlying the variability of the belted coat pattern in the Cinghiato pig population. Genomic analyses revealed several regions of interest within ROH_islands on six autosomes, which may serve as valuable targets to support future selection strategies. Although no specific polymorphisms were identified in the *KIT* and *MC1R* genes to explain the belted phenotype, the presence of variants in other genes suggests a polygenic architecture underlying coat color variability in this population. Given the limited number of animals analyzed, further studies with larger sample sizes are needed to clarify the genetic mechanisms influencing coat color in pigs. More broadly, this work, in line with European strategies for sustainable food systems, contributes to the characterization of a local pig population reconstituted through phenotypic selection. In the context of local resource enhancement, such initiatives may play a role in promoting biodiversity and supporting the developmentof local economies.

## Supplementary Material

Figures_S2.png

FIGURE_S1.png

Table_S1.docx

Table_S2.xlsx

## Data Availability

The 4 WGS Cinghiato pigs (.bam files) were available at NCBI under Sequence Read Archive (SRA) accession numbers PRJNA1174116 (BioSample accessions: SUP_09: SAMN44331701, SUP_16: SAMN44331702, SUP_18: SAMN44331703, SUP_19: SAMN44331704). All other results are included in the Supporting Information.

## References

[1] Sponenberg DP, Martin A, Couch C, Beranger J. Conservation strategies for local breed biodiversity. *Diversity* 2019;11(10):177.

[2] Commission ISS. *IUCN SSC Guiding Principles on Creating Proxies of Extinct Species for Conservation Benefit*. Gland, Switzerland: IUCN Grey Literature; 2016.

[3] Shapiro B. Pathways to de-extinction: how close can we get to resurrection of an extinct species? *Funct Ecol.* 2017;31(5):996–1002.

[4] Stokstad E. Bringing back the aurochs. *Science*. 2015;350(6265):1144–1147. 26785454 10.1126/science.350.6265.1144

[5] Caffarelli M, 3a PTA, Sarti FM, Lasagna E. Suino nero cinghiato. Storia del recupero e della reintroduzione di un’antica popolazione suina in Valnerina. Vol. 4. EDIZIONI 3A-PTA. Regione Umbria Servizio Sviluppo Sostenibile e Gestione Procedure P.S.R; 2014.

[6] Yang B, Cui L, Perez-Enciso M, et al. Genome-wide SNP data unveils the globalization of domesticated pigs. *Genet Sel Evol*. 2017;49(1):71. 28934946 10.1186/s12711-017-0345-y PMC5609043

[7] Wickham H. *ggplot2: Elegant Graphics for Data Analysis*. New York: Springer; 2016.

[8] Ferenčaković M, Sölkner J, Curik I. Estimating autozygosity from high-throughput information: effects of SNP density and genotyping errors. *Genet Sel Evol*. 2013;45(1):42. 24168655 10.1186/1297-9686-45-42 PMC4176748

[9] Mastrangelo S, Sardina MT, Tolone M, et al. Genome-wide identification of runs of homozygosity islands and associated genes in local dairy cattle breeds. *Animal*. 2018;12(12):2480–2488. 29576040 10.1017/S1751731118000629

[10] Schiavo G, Bovo S, Bertolini F, et al. Runs of homozygosity islands in Italian cosmopolitan and autochthonous pig breeds identify selection signatures in the porcine genome. *Livest Sci.* 2020;240:104219.

[11] Barbato M, Orozco-terWengel P, Tapio M, Bruford MW. SNeP: a tool to estimate trends in recent effective population size trajectories using genome-wide SNP data. *Front Genet*. 2015;6:109. 25852748 10.3389/fgene.2015.00109 PMC4367434

[12] Alexander DH, Novembre J, Lange K. Fast model-based estimation of ancestry in unrelated individuals. *Genome Res*. 2009;19(9):1655–1664. 19648217 10.1101/gr.094052.109 PMC2752134

[13] Fontanesi L, D’Alessandro E, Scotti E, et al. Genetic heterogeneity and selection signature at the KIT gene in pigs showing different coat colours and patterns. *Anim Genet*. 2010;41(5):478–492. 20477793 10.1111/j.1365-2052.2010.02054.x

[14] Chen S, Zhou Y, Chen Y, Gu J. fastp: an ultra-fast all-in-one FASTQ preprocessor. *Bioinformatics*. 2018;34(17):i884–i890. 30423086 10.1093/bioinformatics/bty560 PMC6129281

[15] Li H, Durbin R. Fast and accurate short read alignment with Burrows-Wheeler transform. *Bioinformatics*. 2009;25(14):1754–1760. 19451168 10.1093/bioinformatics/btp324 PMC2705234

[16] Li H, Handsaker B, Wysoker A, et al. The sequence alignment/map format and SAM tools. *Bioinformatics*. 2009;25(16):2078–2079. 19505943 10.1093/bioinformatics/btp352 PMC2723002

[17] Wang K, Li M, Hakonarson H. ANNOVAR: functional annotation of genetic variants from high-throughput sequencing data. *Nucleic Acids Res*. 2010;38(16):e164–e164. 20601685 10.1093/nar/gkq603 PMC2938201

[18] Abyzov A, Urban AE, Snyder M, Gerstein M. CNVnator: an approach to discover, genotype, and characterize typical and atypical CNVs from family and population genome sequencing. *Genome Res*. 2011;21(6):974–984. 21324876 10.1101/gr.114876.110 PMC3106330

[19] Muñoz M, Bozzi R, García-Casco J, et al. Genomic diversity, linkage disequilibrium and selection signatures in European local pig breeds assessed with a high density SNP chip. *Sci Rep*. 2019;9(1):13546. 31537860 10.1038/s41598-019-49830-6 PMC6753209

[20] Bordonaro S, Chessari G, Mastrangelo S, et al. Genome-wide population structure, homozygosity, and heterozygosity patterns of Nero Siciliano pig in the framework of Italian and cosmopolitan breeds. *Anim Genet*. 2023;54(5):591–605. 37381662 10.1111/age.13344

[21] Schiavo G, Bovo S, Bertolini F, et al. Comparative evaluation of genomic inbreeding parameters in seven commercial and autochthonous pig breeds. *Animal*. 2020;14(5):910–920. 31928538 10.1017/S175173111900332X

[22] Xie R, Shi L, Liu J, et al. Genome-wide scan for runs of homozygosity identifies candidate genes in three pig breeds. *Animals*. 2019;9(8):518. 31374971 10.3390/ani9080518 PMC6720638

[23] Ramos AM, Crooijmans RPMA, Affara NA, et al. Design of a high density SNP genotyping assay in the pig using SNPs identified and characterized by next generation sequencing technology. *PLoS One*. 2009;4(8):e6524. 19654876 10.1371/journal.pone.0006524 PMC2716536

[24] Meuwissen THE. Accuracy of breeding values of’unrelated’individuals predicted by dense SNP genotyping. *Genet Sel Evol*. 2009;41(1):9. 19519896 10.1186/1297-9686-41-35 PMC2708128

[25] McQuillan R, Leutenegger A-L, Abdel-Rahman R, et al. Runs of homozygosity in European populations. *Am J Hum Genet*. 2008;83(3):359–372. 18760389 10.1016/j.ajhg.2008.08.007 PMC2556426

[26] Bosse M, Megens H-J, Madsen O, et al. Regions of homozygosity in the porcine genome: consequence of demography and the recombination landscape. *PLoS Genet*. 2012;8(11):e1003100. 23209444 10.1371/journal.pgen.1003100 PMC3510040

[27] Scandura M, Iacolina L, Crestanello B, et al. Ancient vs. recent processes as factors shaping the genetic variation of the European wild boar: are the effects of the last glaciation still detectable? *Mol Ecol*. 2008;17(7):1745–1762. 18371016 10.1111/j.1365-294X.2008.03703.x

[28] Derks MFL, Gross C, Lopes MS, et al. Accelerated discovery of functional genomic variation in pigs. *Genomics.* 2021;113(4):2229–2239. 34022350 10.1016/j.ygeno.2021.05.017

[29] Onteru SK, Fan B, Du Z, Garrick DJ, Stalder KJ, Rothschild MF. A whole‐genome association study for pig reproductive traits. *Anim Genet*. 2012;43(1):18–26. 22221021 10.1111/j.1365-2052.2011.02213.x

[30] Szmatoła T, Jasielczuk I, Semik-Gurgul E, et al. Detection of runs of homozygosity in conserved and commercial pig breeds in Poland. *J Anim Breed Genet*. 2020;137(6):571–580. 32362048 10.1111/jbg.12482

[31] Feng X, Diao S, Liu Y, et al. Exploring the mechanism of artificial selection signature in Chinese indigenous pigs by leveraging multiple bioinformatics database tools. *BMC Genomics*. 2023;24(1):743. 38053015 10.1186/s12864-023-09848-7 PMC10699062

[32] Le TH, Christensen OF, Nielsen B, Sahana G. Genome-wide association study for conformation traits in three Danish pig breeds. *Genet Sel Evol*. 2017;49(1):12. 28118822 10.1186/s12711-017-0289-2 PMC5259967

[33] Wang F, Zha Z, He Y, et al. Genome-wide re-sequencing data reveals the population structure and selection signatures of Tunchang pigs in China. *Animals*. 2023;13(11):1835. 37889708 10.3390/ani13111835 PMC10252034

[34] Lee S-H, Lee S-H, Park H-B, Kim J-M. Identification of key adipogenic transcription factors for the pork belly parameters via the association weight matrix. *Meat Sci*. 2023;195:109015. 36334514 10.1016/j.meatsci.2022.109015

[35] Lim K, Cho S-I, Kim J-S. Nuclear and mitochondrial DNA editing in human cells with zinc finger deaminases. *Nat Commun*. 2022;13(1):366. 35042880 10.1038/s41467-022-27962-0 PMC8766470

[36] Vahedi SM, Salek Ardestani S, Karimi K, Banabazi MH. Weighted single-step GWAS for body mass index and scans for recent signatures of selection in Yorkshire pigs. *J Hered*. 2022;113(3):325–335. 35079818 10.1093/jhered/esac004

[37] Gòdia M, Estill M, Castelló A, et al. A RNA-seq analysis to describe the boar sperm transcriptome and its seasonal changes. *Front Genet*. 2019;10:299. 31040860 10.3389/fgene.2019.00299 PMC6476908

[38] Aare S, Radell P, Eriksson LI, Chen Y-W, Hoffman EP, Larsson L. Role of sepsis in the development of limb muscle weakness in a porcine intensive care unit model. *Physiol Genomics*. 2012;44(18):865–877. 22851759 10.1152/physiolgenomics.00031.2012

[39] Dawson HD, Smith AD, Chen C, Urban JF. An in-depth comparison of the porcine, murine and human inflammasomes; lessons from the porcine genome and transcriptome. *Vet Microbiol*. 2017;202:2–15. 27321134 10.1016/j.vetmic.2016.05.013

[40] Qian R, Xie F, Zhang W, et al. Genome-wide detection of CNV regions between Anqing six-end-white and Duroc pigs. *Mol Cytogenet*. 2023;16(1):12. 37400846 10.1186/s13039-023-00646-0 PMC10316616

[41] Crespo-Piazuelo D, Acloque H, González-Rodríguez O, et al. Identification of transcriptional regulatory variants in pig duodenum, liver, and muscle tissues. *GigaScience*. 2022;12:giad042. 37354463 10.1093/gigascience/giad042 PMC10290502

[42] Li X, Yan Z, Ma J, et al. TRIM28 promotes porcine epidemic diarrhea virus replication by mitophagy-mediated inhibition of the JAK-STAT1 pathway. *Int J Biol Macromol*. 2024;254(Pt 1):127722. 37907173 10.1016/j.ijbiomac.2023.127722

[43] Liu A, Yang Y, Guo J, et al. Cytochrome P450 enzymes mediated by DNA methylation is involved in deoxynivalenol-induced hepatoxicity in piglets. *Anim Nutr*. 2022;9:269–279. 35600548 10.1016/j.aninu.2021.11.009 PMC9092380

[44] Arias KD, Gutiérrez JP, Fernández I, Álvarez I, Goyache F. Copy number variation regions differing in segregation patterns span different sets of genes. *Animals*. 2023;13(14):2351. 37508128 10.3390/ani13142351 PMC10376189

[45] Wang Z, Zhong Z, Xie X, et al. Detection of runs of homozygosity and identification of candidate genes in the whole genome of Tunchang pigs. *Animals*. 2024;14(2):201. 38254370 10.3390/ani14020201 PMC10812771

[46] París-Oller E, Navarro-Serna S, Soriano-Úbeda C, et al. Reproductive fluids, used for the in vitro production of pig embryos, result in healthy offspring and avoid aberrant placental expression of PEG3 and LUM. *J Animal Sci Biotechnol*. 2021;12(1):32. 10.1186/s40104-020-00544-0 PMC7883450 33583428

[47] Lim K-S, Lee K-T, Lee S-W, et al. Genomic structure, expression and association study of the porcine FSD2. *Mol Biol Rep*. 2016;43(9):1011–1018. 27350214 10.1007/s11033-016-4029-4

[48] Zhang L, Zhang S, Zhan F, et al. Population genetic analysis of six Chinese indigenous pig meta-populations based on geographically isolated regions. *Animals*. 2023;13(8):1396. 37106959 10.3390/ani13081396 PMC10135051

[49] Reyer H, Varley PF, Murani E, Ponsuksili S, Wimmers K. Genetics of body fat mass and related traits in a pig population selected for leanness. *Sci Rep*. 2017;7(1):9118. 28831160 10.1038/s41598-017-08961-4 PMC5567295

[50] Zhang H, Zhuang Z, Yang M, et al. Genome-wide detection of genetic loci and candidate genes for body conformation traits in Duroc × Landrace × Yorkshire crossbred pigs. *Front Genet*. 2021;12:664343. 34707635 10.3389/fgene.2021.664343 PMC8542986

[51] Dong K, Yao N, Pu Y, et al. Genomic scan reveals loci under altitude adaptation in Tibetan and Dahe pigs. *PLoS One*. 2014;9(10):e110520. 25329542 10.1371/journal.pone.0110520 PMC4201535

[52] Keel BN, Snelling WM, Lindholm-Perry AK, Oliver WT, Kuehn LA, Rohrer GA. Using SNP weights derived from gene expression modules to improve GWAS power for feed efficiency in pigs. *Front Genet*. 2019;10:1339. 32038708 10.3389/fgene.2019.01339 PMC6985563

[53] Bovo S, Ribani A, Muñoz M, et al. Whole-genome sequencing of European autochthonous and commercial pig breeds allows the detection of signatures of selection for adaptation of genetic resources to different breeding and production systems. *Genet Sel Evol*. 2020;52(1):33. 32591011 10.1186/s12711-020-00553-7 PMC7318759

[54] Snyder E, Chukrallah L, Seltzer K, Goodwin L, Braun RE. ADAD1 and ADAD2, testis-specific adenosine deaminase domain-containing proteins, are required for male fertility. *Sci Rep*. 2020;10(1):11536. 32665638 10.1038/s41598-020-67834-5 PMC7360552

[55] Urs S, Venkatesh D, Tang Y, et al. Sprouty1 is a critical regulatory switch of mesenchymal stem cell lineage allocation. *FASEB J*. 2010;24(9):3264–3273. 20410440 10.1096/fj.10-155127 PMC2923355

[56] Addo S, Jung L. An insight into the runs of homozygosity distribution and breed differentiation in Mangalitsa pigs. *Front Genet*. 2022;13:909986. 36338970 10.3389/fgene.2022.909986 PMC9632489

[57] Pugliese C, Bozzi R, Gallo M, et al. Cinta Senese Pig. European Local Pig Breeds - Diversity and Performance*. A Study of Project TREASURE*. London: IntechOpen; 2019.

[58] Tinarelli S, Ribani A, Utzeri VJ, et al. Redefinition of the Mora Romagnola pig breed herd book standard based on DNA markers useful to authenticate its “mono-breed” products: an example of sustainable conservation of a livestock genetic resource. *Animals.* 2021;11(2):526. 33670521 10.3390/ani11020526 PMC7923016

[59] Poklukar K, Mestre C, Škrlep M, et al. A meta-analysis of genetic and phenotypic diversity of European local pig breeds reveals genomic regions associated with breed differentiation for production traits. *Genet Sel Evol*. 2023;55(1):88. 38062367 10.1186/s12711-023-00858-3 PMC10704730

[60] Zhang C, Plastow G. Genomic diversity in pig (*Sus scrofa*) and its comparison with human and other livestock. *Curr Genomics*. 2011;12(2):138–146. 21966252 10.2174/138920211795564386 PMC3129048

[61] Laval G, Iannuccelli N, Legault C, et al. Genetic diversity of eleven European pig breeds. *Genet Sel Evol*. 2000;32:187–203. 14736401 10.1186/1297-9686-32-2-187 PMC2706869

[62] Qin M, Li C, Li Z, Chen W, Zeng Y. Genetic diversities and differentially selected regions between shandong indigenous pig breeds and western pig breeds. *Front Genet*. 2019;10:1351. 32038711 10.3389/fgene.2019.01351 PMC6987402

[63] Fontanesi L. Invited review: genetics and genomics of pigmentation variability in pigs: a review. *Livest Sci.* 2022;265:105079.

[64] Koseniuk A, Smołucha G, Natonek-Wiśniewska M, Radko A, Rubiś D. Differentiating pigs from wild boars based on NR6A1 and MC1R gene polymorphisms. *Animals.* 2021;11(7):2123. 34359251 10.3390/ani11072123 PMC8300376

[65] Wu Z, Deng Z, Huang M, et al. Whole-genome ­resequencing identifies KIT new alleles that affect coat color phenotypes in pigs. *Front Genet*. 2019;10:218. 30949195 10.3389/fgene.2019.00218 PMC6436083

[66] Giuffra E, Evans G, Törnsten A, et al. The Belt mutation in pigs is an allele at the Dominant white (I/KIT) locus. *Mamm Genome*. 1999;10(12):1132–1136. 10594235 10.1007/s003359901178

[67] Xu J, Fu Y, Hu Y, et al. Whole genome variants across 57 pig breeds enable comprehensive identification of genetic signatures that underlie breed features. *J Anim Sci Biotechnol*. 2020;11(1):115. 33292532 10.1186/s40104-020-00520-8 PMC7713148

[68] Bovo S, Ribani A, Muñoz M, et al. Genome‐wide detection of copy number variants in European autochthonous and commercial pig breeds by whole‐genome sequencing of DNA pools identified breed‐characterising copy number states. *Anim Genet*. 2020;51(4):541–556. 32510676 10.1111/age.12954

[69] Jin L, Zhao L, Hu S, et al. Transcriptional differences of coding and non-coding genes related to the absence of melanocyte in skins of Bama pig. *Genes*. 2019;11(1):47. 31905971 10.3390/genes11010047 PMC7017308

[70] Lin T, Luo L, Guo W, et al. Phenotypic similarities in pigs with *SOX10*c.321dupC and *SOX10*c.325A > T mutations implied the correlation of SOX10 haploinsufficiency with Waardenburg syndrome. *J Genet Genomics*. 2020;47(12):770–780. 33766494 10.1016/j.jgg.2020.12.003

[71] Qi J-C, Jiang Q-Q, Ma L, et al. Sox10 gene is required for the survival of saccular and utricular hair cells in a porcine model. *Mol Neurobiol*. 2022;59(6):3323–3335. 35249166 10.1007/s12035-021-02691-5

[72] Fernández A, Silió L, Rodríguez C, Óvilo C. Characterization of OCA2 cDNA in different porcine breeds and analysis of its potential effect on skin pigmentation in a red Iberian strain. *Anim Genet*. 2006;37(2):166–170. 16573532 10.1111/j.1365-2052.2005.01393.x

[73] Xu Z, Wu J, Zhang Y, et al. Genome-wide detection of selection signatures in Jianli pigs reveals novel cis-regulatory haplotype in EDNRB associated with two-end black coat color. *BMC Genomics*. 2024;25(1):23. 38166718 10.1186/s12864-023-09943-9 PMC10763394

[74] Gallozzi F. First documented observation of differential dorsoventral coat colouration in wild boar *Sus scrofa* (Artyodactyla: Suidae) in Italy. *Nat Hist Sci*. 2024;11(1):71–72.

[75] Wang C, Wang H, Zhang Y, Tang Z, Li K, Liu B. Genome‐wide analysis reveals artificial selection on coat colour and reproductive traits in C hinese domestic pigs. *Mol Ecol Resour*. 2015;15(2):414–424. 25132237 10.1111/1755-0998.12311

