# Supplementary figures and images for: Genomic characterization and insights into the belted coat pattern of a local, reconstituted pig population
Source: Anim Biotechnol. 2025 Jun 10;36(1):2515462. doi: 10.1080/10495398.2025.2515462 (PMC12674266; doi:10.1080/10495398.2025.2515462)

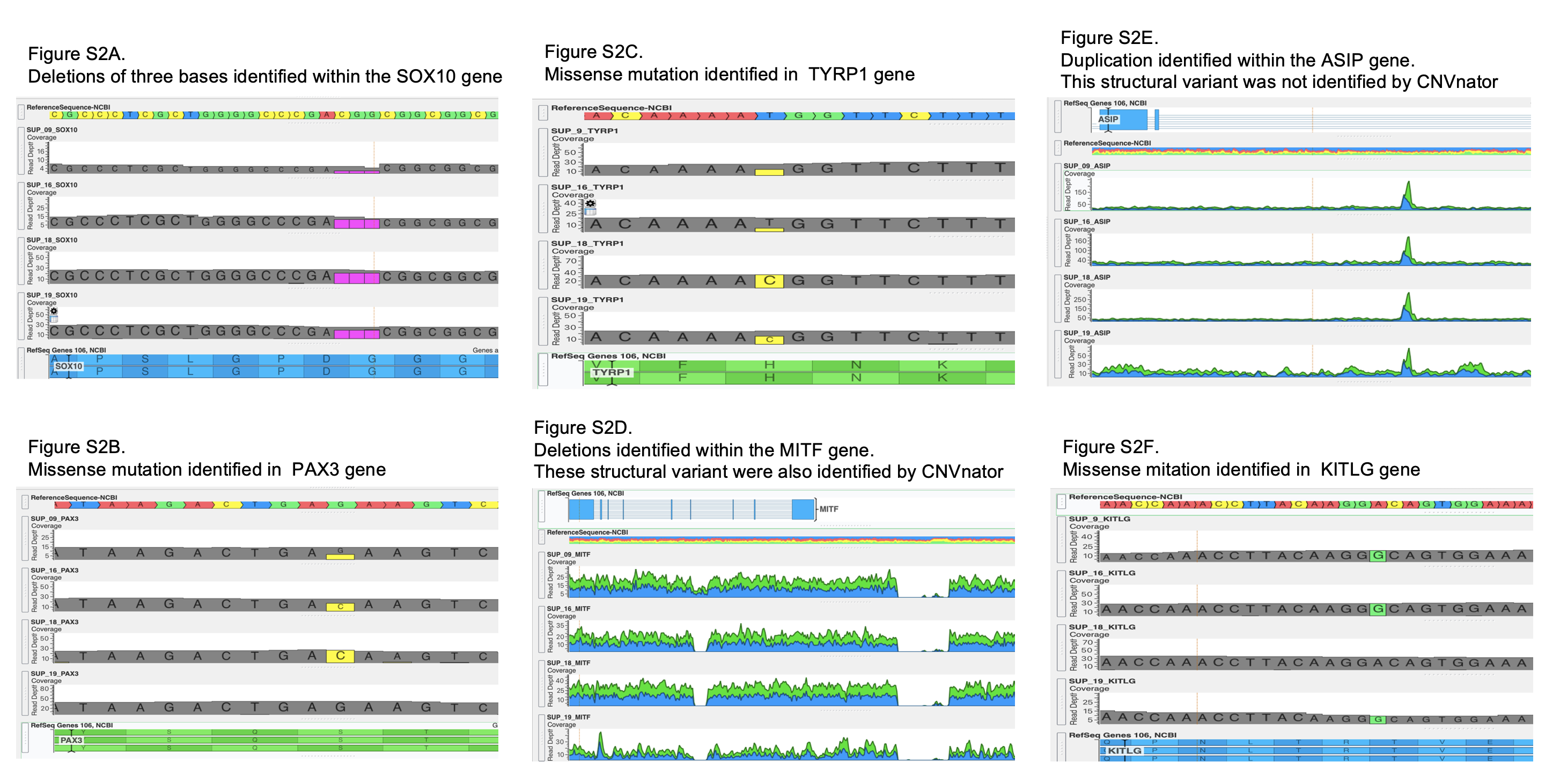

Supplement: Figures_S2.png [file LABT_A_2515462_SM5528.png]

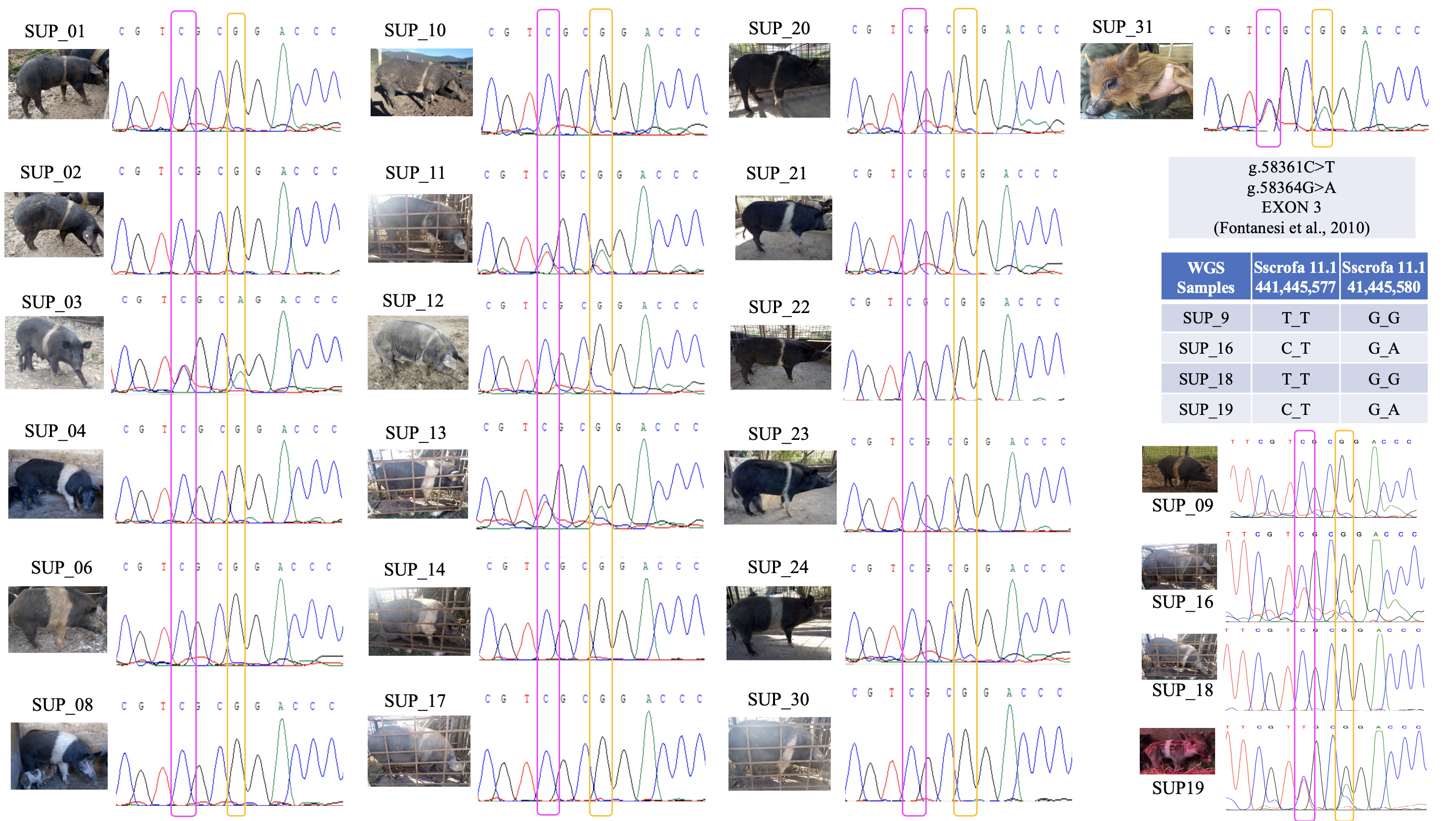

Supplement: FIGURE_S1.png [file LABT_A_2515462_SM5527.png]
