# Supplementary material for: Genomic characterization and insights into the belted coat pattern of a local, reconstituted pig population
Source: Anim Biotechnol. 2025 Jun 10;36(1):2515462. doi: 10.1080/10495398.2025.2515462 (PMC12674266; doi:10.1080/10495398.2025.2515462)
Supplement: Table_S1.docx [file LABT_A_2515462_SM5526.docx]

**Table S1.** *MC1R* and *KIT* gene exons features according with GCF_000003025.6_Sscrofa11.1 genome assembly

| **Exon** | **Size** | **Start** | **End** | **Note** |
| --- | --- | --- | --- | --- |
| ***MC1R*** (Chr 6; NC_010448.4) | | | | |
| 1 | 963 | 181,225 | 182,188 |  |
| ***KIT*** (chr 8; NC_010450.4) | | | | |
| 1 | 96 | 41,402,334 | 41,402,430 | Include 5’UTR |
| 2 | 270 | 41,441,331 | 41,441,601 |  |
| 3 | 282 | 41,445,426 | 41,445,708 |  |
| 4 | 137 | 41,446,769 | 41,446,906 |  |
| 5 | 62 | 41,451,326 | 41,451,388 |  |
| 6 | 104 | 41,451,390 | 41,451,494 |  |
| 7 | 190 | 41,456,866 | 41,457,056 |  |
| 8 | 116 | 41,459,172 | 41,459,288 |  |
| 9 | 115 | 41,474,707 | 41,474,822 |  |
| 10 | 182 | 41,477,863 | 41,478,045 |  |
| 11 | 107 | 41,479,445 | 41,479,552 |  |
| 12 | 127 | 41,479,647 | 41,479,774 |  |
| 13 | 105 | 41,480,340 | 41,480,445 |  |
| 14 | 111 | 41,480,529 | 41,480,640 |  |
| 15 | 151 | 41,481,899 | 41,482,050 |  |
| 16 | 92 | 41,484,087 | 41,484,179 |  |
| 17 | 128 | 41,484,668 | 41,484,796 |  |
| 18 | 123 | 41,485,889 | 41,486,012 |  |
| 19 | 112 | 41,488,443 | 41,488,555 |  |
| 20 | 100 | 41,488,670 | 41,488,770 |  |
| 21 | 106 | 41,489,407 | 41,489,513 |  |
| 22 | 903 | 41,491,404 | 41,492,307 | Include 3’UTR |
